# Prognostic Factors for Overall Survival in Nasopharyngeal Cancer and Implication for TNM Staging by UICC: A Systematic Review of the Literature

**DOI:** 10.3389/fonc.2021.703995

**Published:** 2021-09-02

**Authors:** Chi Leung Chiang, Qiaojuan Guo, Wai Tong Ng, Shaojun Lin, Tiffany Sze Wai Ma, Zhiyuan Xu, Youping Xiao, Jishi Li, Tianzhu Lu, Horace Cheuk Wai Choi, Wenqi Chen, Eric Sze Chun Chau, Peter Ho Yin Luk, Shao Hui Huang, Brian O’Sullivan, Jianji Pan, Anne Wing Mui Lee

**Affiliations:** ^1^Clinical Oncology Center, The University of Hong Kong-Shenzhen Hospital, Shenzhen, China; ^2^Department of Clinical Oncology, Li Ka Shing Faculty of Medicine, The University of Hong Kong, Hong Kong, Hong Kong; ^3^Department of Radiation Oncology, Fujian Cancer Hospital & Fujian Medical University Cancer Hospital, Fuzhou, China; ^4^Department of Radiation Oncology, University of Toronto, Princess Margaret Cancer Centre, Toronto, ON, Canada

**Keywords:** nasopharyngeal carcinoma, prognostic factors, AJCC/UICC staging system, TMN classification, systematic review, anatomical criteria

## Abstract

This study aims to identify prognostic factors in nasopharyngeal carcinoma (NPC) to improve the current 8th edition TNM classification. A systematic review of the literature reported between 2013 and 2019 in PubMed, Embase, and Scopus was conducted. Studies were included if (1) original clinical studies, (2) ≥50 NPC patients, and (3) analyses on the association between prognostic factors and overall survival. The data elements of eligible studies were abstracted and analyzed. A level of evidence was synthesized for each suggested change to the TNM staging and prognostic factors. Of 5,595 studies screened, 108 studies (44 studies on anatomical criteria and 64 on non-anatomical factors) were selected. Proposed changes/factors with strong evidence included the upstaging paranasal sinus to T4, defining parotid lymph node as N3, upstaging N-category based on presence of lymph node necrosis, as well as the incorporation of non-TNM factors including EBV-DNA level, primary gross tumor volume (GTV), nodal GTV, neutrophil-lymphocyte ratio, lactate dehydrogenase, C-reactive protein/albumin ratio, platelet count, SUVmax of the primary tumor, and total lesion glycolysis. This systematic review provides a useful summary of suggestions and prognostic factors that potentially improve the current staging system. Further validation studies are warranted to confirm their significance.

## Introduction

Nasopharyngeal carcinoma (NPC) is an important global health burden with approximately 130,000 new cases diagnosed and more than 70,000 deaths in 2018 (1). It is a unique disease with distinctive natural behavior, epidemiology, and histopathology that differs from other head and neck cancers. Estimation of prognosis is a fundamental step in patient management. Among the various prognostic factors, the tumor–node–metastasis (TNM) staging, which has been jointly adopted by the American Joint Committee on Cancer (AJCC) and the Union for International Cancer Control (UICC), remains the most robust factor for global application. The TNM 5^th^ Edition issued in 1997, which introduced a customized staging system for NPC by merging the strengths of the AJCC/UICC 4^th^ edition and Ho’s system, is a historic milestone with worldwide acceptance. Subsequent revisions refined the staging system based on diagnostic and therapeutic advances (2, 3); the current 8^th^ Edition, released in 2017, is another milestone with the unification of the TNM and the Chinese staging systems (4).

In addition to the refinement of TNM parameters, there is a growing interest in the incorporation of non-anatomical prognostic factors that reflect biological tumor behavior. These factors are potentially useful for providing biomarkers on personalized risk stratification, especially with regard to metastatic risk, for tailoring the treatment intensity. There is increasing evidence that incorporation of these factors/biomarkers with TNM staging system could further improve risk stratification (5, 6).

To provide the best available evidence for the upcoming TNM 9^th^ Edition and associated prognostic grouping, a comprehensive systematic review was carried out to identify potentially important suggestions on anatomic and non-anatomic prognostic factors. These suggestions will then be confirmed by a multicenter validation study before the final recommendation to UICC and AJCC for consideration. The current paper is our summary of suggested prognostic factors that warrant further validation.

## Materials and Methods

### Study Protocol

This review adhered to the Preferred Reporting Items for Systematic Reviews and Meta-Analysis (PRISMA) guideline (7). A systematic search of PubMed, Scopus, and Embase for relevant literature published from January 1, 2013, to September 13, 2019, was performed. This timeframe was selected because the construction of TNM 8^th^ Edition was based on literature reviews up to December 31, 2012. Both English and Chinese literatures were accepted, although unpublished studies were not included in the search. The search terms (**Supplementary Table 1**) were as follows: (“staging” or “TNM” or “prognostic”) and (“nasopharyngeal carcinoma” or “nasopharyngeal cancer” or “nasopharyngeal neoplasm”).

### Inclusion Process and Criteria

From the literature identified in the initial search, the following studies were excluded after screening their titles and citations: duplicated studies, conference abstracts, reviews, letters, editorials, case reports, book chapters, and basic science studies. The remaining studies were further assessed to determine eligibility, which included original clinical studies, either prospective or retrospective, with a sample size of at least 50 NPC patients, treated with intensity modulated radiotherapy (IMRT) or equivalent, and showing a significant association between prognostic factors and overall survival (OS). Novel prognostic markers with limited potential for global applicability (e.g., radiomics, micro-RNA, circulating tumor cells, and genetic signatures) were excluded from this review. In cases of multiple studies from one institution, the study with the largest number of patients and the most recently published study was prioritized.

Two independent teams (University of Hong Kong–Shenzhen Hospital and Fujian Cancer Hospital) performed the first review to exclude the ineligible studies. Three independent reviewers (AL, W-TN, and C-LC) further assessed papers that generated disagreements based on the inclusion/exclusion before a final decision was made on the list of studies to be selected for inclusion in this review.

### Data Extraction and Analyses

The primary data from the articles were extracted. The primary endpoint for the assessment of prognostic value in this review was OS; the secondary endpoints of distant-metastasis-free survival (DMFS) and local-relapse-free survival (LRFS) were included if they were reported by the original study.

We used the QUality In Prognosis Studies (QUIPS) tool to assess the risk of bias within individual studies (8). The QUIPS tool was originally designed to assess bias in studies of prognostic factors. The tool originally comprised six domains—Study Participation, Prognostic Factor Measurement, Outcome Measurement, Statistical Analysis and Reporting, Study Confounding, and Study Attrition—each of which is guided by three to seven prompting items. Based on the risk of bias, the overall quality of each study was determined as high (score 5–6), moderate (score 3–4), or low (score 0–2); low-quality studies were excluded from this review.

The criteria adopted in this systematic review were designed to synthesize the level of evidence (9), which was defined as “strong,” if there were consistent recommendations (≥75%) in multiple high-quality cohorts; “moderate,” if recommendations were consistent in ≥67% of multiple high-quality cohorts; “limited,” if the recommendation was based on a single cohort; and “inconclusive,” if there were inconsistent recommendations.

## Results

### Study Selection

An initial search of the three databases identified 5,595 studies that fit the search terms. Following the exclusion of ineligible articles (based on the predefined study eligibility criteria), two independent teams were constituted to identify new suggestions for improving the current TNM 8^th^ Edition. Among the 2,200 studies evaluated, 34 original studies were selected for inclusion by both teams, whereas 198 studies were selected by only one team. The studies with a discrepancy in agreement were further reviewed by three independent reviewers, and 74 were accepted for inclusion. Thus, a total of 108 original studies were included in this in-depth systematic review.

### Study Characteristics

The characteristics of the 108 studies are presented in **Supplementary Tables 2–6**. Only six studies are prospective analyses, while the rest (n = 102) were retrospective. The majority of studies (n = 101) included only patients without distant metastasis. Forty-four studies focused on anatomical criteria: 22 studies on primary tumor (T-classification) (6, 10–30), 20 on nodal disease (N-classification) (14, 22, 23, 31–47), 5 studies on metastatic disease (M-classification) (48–52), and 3 studies included more than one category. In the 64 studies that evaluated non-anatomical factors, 22 studies focused on plasma Epstein-Barr virus (EBV) deoxyribonucleic acid (DNA) level (53–71), 12 studies on tumor volume (63, 65, 72–81), 18 studies on inflammatory/hematological factors (54, 82–98), and 15 studies on the parameters of fluorodeoxyglucose (FDG) positron emission tomography (PET) (99–113). Three studies had more than one non-anatomical category.

### Risks of Bias

The assessment on study quality using the QUIPS tool showed that 62 (57.4%) of the included articles were classified as high quality and 46 (42.6%) as moderate quality. **Supplementary Figure 1** presents an algorithm of the study selection process, and **Supplementary Tables 2–6** list the QUIPS scores of the included studies. Suggestions from well-conducted studies with large sample sizes or with evidence supported by multiple studies were identified for inclusion in this review.

### Proposed Changes and Prognostic Factors

Summary of the level of evidence on the recommendations and studied prognostic factors is summarized in **Table 1**. Among the 44 reports on TNM parameters (**Table 2**), 13 proposed changes to current TNM-8 were identified: six on T-category, eight on N-category, and one on M-category. The recommendations that were considered to have a strong level of evidence included the involvement of the paranasal sinus (PNS) as T4 disease (16, 18–20), parotid lymph node (PLN) as N3 disease (34, 35), and the upstaging of N-classification in the presence of lymph node necrosis (LNN) (39, 40, 42).

**Table 1:** Proposed prognostic factors and the level of evidence for recommendations.

	Strong^*^	Moderate^*^	Inconclusive^*^	Limited^*^
T-classification	PNS involvement (T4)		MP (upstaged to T3/4)	Skull base (T3 slight *vs.* T3 severe)
			LP (upstaged to T3/4)	Intra-cranial extension (T4a *vs.* T4b)
			Merging of T-classification (Merging of T1/T2, T1/T2/T3, or T2/3)	
N-classification	PLN (N3)	ENE (upstaging of N- classification or N3)	RLN	PLV LN
	LNN (upstaging of N- classification)			Cervical LN level
				Number of LN regions
				Merging of N classification (N2/N3)
M-classification			Subclassification of M-stage (based on number of lesions, number of organ involvement, liver involvement)	
EBV-DNA level	Pretreatment EBV-DNA level			
	(Range: 1,500–25,000 copies/ml)			
Tumor volume	Primary GTV volume (Range: 20–50 ml)			
	Nodal GTV volume (Range: 7.2–35.7 ml)			
Inflammatory or hematological markers	NLR (Range: 2.28–3.0)			Hs-CRP
	CRP/albumin ratio (Range: 0.03–0.141)			PNI and AGR
	Anemia (Range: 11–13 g/dl)			D-dimer
	Platelet count (Range: 266–300 × 10^9^/L)			TIL
	LDH (Range: 220–229 U/L)			PDW
FDG-PET parameters	SUV_max_ of the primary tumor (Range: 8–18.8)		SUV_max_ of nodal disease	MTV
	TLG (Range: 322.7–382.2)		SUV_max_ of metastatic disease	

PNS, paranasal sinus; IF, infratemporal fossa; PM, prevertebral muscle; MP, medial pterygoid; LP, lateral pterygoid; PLN, parotid lymph node; LNN, lymph node necrosis; ENE, extra-nodal extension; PLV LN, posterior to level V lymph node; LN, lymph node; RLN, retropharyngeal lymph node; EBV-DNA, Epstein-Barr virus deoxyribonucleic acid; GTV, gross tumor volume; NLR, neutrophil-lymphocyte ratio; CRP, C-reactive protein; PDW, platelet distribution width; LDH, lactate dehydrogenase; Hs-CRP, high-sensitivity CRP; PNI, prognostic nutrition index; AGR, albumin/globulin ratio; TIL, tumor-infiltrating lymphocytes; SUV_max_, maximum standardized uptake ratio; TLG, total lesion glycolysis; MTV, metabolic tumor volume.

*Level of evidence: “strong,” if there were consistent recommendations (≥75%) in multiple high-quality cohorts; “moderate,” if recommendations were consistent in ≥67% of multiple high-quality cohorts; “limited,” if the recommendation was based on a single cohort; and “inconclusive,” if there were inconsistent recommendations.

**Table 2:** Characteristics of studies of T-, N-, and M-classification prognostic factors and survival (n=44).

(I) T-classification (n=22)											
Study(Author/Year of publication)	Study design(P/R)	Sample size	TNM/UICC staging	Prognostic factor	HR (high vs. low)	95% CI	*P* value	Survival probabilities	*P* value	Proposed changes	Quality score
**(A) Medial pterygoid (MP), lateral pterygoid (LP), prevertebral muscle (PM), infratemporal fossa (IF), and others**											
Luo (2014) (10)	R	742	7^th^ Edition Stage III–IVB	MP or LP (Yes vs. No)	1.658	1.058–2.596	NA	5-year OS: 82.5% vs.70.9%	0.001	MP/LP should be staged as T3	5
Sze (2014) (11)	R	1104 (all) 434 (T3)	7^th^ Edition Stage I–IVB	MP ± LP	1.16	0.76–1.76	0.49	–	–	MP ± LP should be classified as T2	5
Zhang (2014) (30)	R	808	7^th^ Edition Stage I–IVB	Masticator space involvement	1.309	1.061–1.615	0.012	–	–	Medial involvement to be classified as T2; lateral involvement to be classified as T4	4
Xiao Y, Pan J, Chen Y (2015) (12)	R	816	7^th^ Edition Stage I–IVB	MP	1.572	1.191–2.074	0.007	5-year OS: w/MP: 69.6%	–	MP similar to T2 prognosis	4
Zhang (2015)(13)	R	1504	7^th^ Edition Stage I–IVB	MP	0.623	0.445–0.873	0.006	–	–	T2: Mild invasion (involvement of medial pterygoid muscle of masticator space or pre-styloid, carotid, prevertebral, or retropharyngeal spaces) T4: extensive invasion (involvement of lateral pterygoid muscle and beyond the masticator space or parotid space).	3
				LP	1.572	1.143–2.163	0.005	–	–		
				Posterior carotid space	1.085	0.787–1.497	0.618	–	–		
				Parapharyngeal extension	1.169	0.756–1.809	0.482	–	–		
Pan (2016) (14)	R	1609	7^th^ Edition Stage I–IVB	MP/LP/PM	1.008	1.004–1.013	<0.001	–	–	MP/LP/PM as T2	6
Zhou (2017) (16)	R	358	7^th^ Edition Stage III–IVb	MP, LP, PNS, skull base, cavernous sinus, CN	–	–	–	5-year OS: Without MP vs. with MP: 86.9% vs. 83.1% without LP vs with LP: 67% vs. 81.6%	<0.001	T3: MP and skull base T4: LP, PNS, MS beyond LP, cavernous sinus, and CN	5
Kang M, Zhou P, Liao X (2017) (15)	R	608	8^th^ Edition Stage I–IVB	MP/LP	3.410	2.016–5.766	<0.001	5-year OS: 75.6% vs. 86.7%	0.043	LP should be graded as T4 MP: T2	4
**(B) Skull base involvement**											
Li (2019) (17)	R	1225	8^th^ Edition Stage I–IVB	Skull base (T3 slight: pterygoid process and/or base of pterygoid bone vs. T3 severe)	1.117	0.557–2.241	0.775	5-year OS: 93.0% vs. 83.5%	0.014	Patients with **T3 slight** (base of pterygoid bone and pterygoid process) should be T2; **T3 severe** remains as T3	6
**(C) Paranasal sinus (PNS) involvement**											
Zhang (2016) (18)	R	1811	7^th^ Edition Stage I–IVB	PNS	2.614	1.455–4.695	0.001	–	–	T3: Sphenoid sinus T4: Ethmoid and maxillary sinuses	5
Zhou (2017) (16)	R	358	7^th^ Edition Stage III–IVb	MP, LP, PNS, skull base, cavernous sinus, CN	–	–	–	5-year OS: 66.3% vs. 84.1%	<0.001	T3: MP and skull base T4: LP, PNS, MS beyond LP, cavernous sinus, and CN	5
Wang Y, Zhao J, Zhao Y (2018) (19)	R	295	8^th^ Edition T3–4, M0	PNS	1.919	1.128–3.264	0.016	5-year OS: 53.7% vs. 80.4%	0.001	PNS to be moved to T4	3
Cao (2019) (20)	R	695	8^th^ Edition Stage I–IVb	PNS	–	–	–	5-year OS: 83.7% vs. 92.2%	0.011	PNS to be reclassified to T4	5
**(D) Intracranial extension or cranial nerve involvement**											
Zong (2014) (28)	R	375	7^th^ Edition Stage III–IVB	Cranial nerve involvement	–	–	–	5-year OS: with MRI-detected CN 71.9% vs. without MRI-detected CN 77.7%	0.134	MRI-detected CN should not be reclassified as T4 to avoid excessive treatment	4
Cao (2017) (29)	R	335	7^th^ Edition T4	Intracranial invasion	0.572	0.389–0.839	0.004	1- / 3- / 5-year: T4a: 95.9% / 83.1% / 71.5% vs. T4b: 91.2% / 69.7% / 51.6%	–	To subclassify T4: (a) without intracranial invasion, (b) with intracranial invasion	5
**(E) Simplification of T-classification**											
Zong (2015) (21)	R	1241	7^th^ Edition Stage I–IVB	T1 vs. T2 vs. T3	1.792	1.295–2.48	<0.001	5-year OS: 88.1%		Merge T1–T2	4
Kang (2016) (22)	R	492	7^th^ Edition Stage I–IVB	T1 vs. T2 vs. T3 vs. T4	1.657	1.101–2.495	0.016	5-year OS: 100% vs 98.2% vs 97.9% vs 88.2%	0.001	Merge T1–T3 to T1, T4 as T2	4
Liang (2016) (23)	R	752	7^th^ Edition Stage I–IVB	T1 vs. T2 vs. T3 vs. T4	–	–	–	–	–	Merge T1–T2 to T1, T3 as T2, T4 as T3	4
Kang M, Zhou P, Wei T (2017) (26)	P	492	7^th^ Edition Stage I–IVB	T1 vs. T2 vs. T3 vs. T4	–	–	–	–	–	T1 (nasopharynx, nasal cavity, parapharyngeal space, oropharynx, skull base, and MP); T2 (LP, paranasal sinus, infratemporal fossa, orbit, cranial nerves, cavernous sinus, and intracalvarium)	5
Li (2018) (24)	R	382	7^th^ Edition Stage I–IVB	T1 vs. T2 vs. T3 vs. T4	2.366	1.685–3.322	<0.001	5-year OS: 92.4% vs. 92.0% vs. 87.3% vs. 76.5%	0.001	Merge T1–T2	6
Yang (2018) (25)	R	1317	7^th^/8^th^ Edition Stage I–IVB	T1 vs. T2 vs. T3 vs. T4	–	–	–	–	–	Refinement of T2–T4 needed as no difference in LRFS in T2–T4; and OS difference in T2 and T3	6
Pan (2019) (6)	R	325	8^th^ Edition Stage I–IVB	T1-T4	–	–	–	–	–	Simplification of the definition of T1, T2, T3, and T4	4
Tang (2019) (27)	R	2191 (training set); 414 (validation set)	8^th^ Edition Stage I–IVB	Merging T2 and T3 (proT1, proT2, and proT3)	proT2: 1.379 proT3: 2.644	pro T2: 0.896–2.121 proT3: 1.667–4.193	–	5-year OS: 93.8% vs. 87.5% vs. 76.0%	<0.001	Merging of T2 and T3 to proposed T2 (proT2)	6
**(II) N-classification (n=19)**											
**Study(Author/Year of publication)**	**Study design(P/R)**	**Sample size**	**TNM/UICC staging**	**Prognostic factor**	**HR (high vs. low)**	**95% CI**	***P* value**	**Survival probabilities**	***P* value**	**Proposed changes**	**Quality score**
**(A) Retropharyngeal LN (RPLN)**											
Shi (2014) (31)	R	142	7^th^ Edition N1 disease	CLN+RLN	–	–	–	5-year OS: 85.3% vs. 95.1%	0.119	Better prognosis: CLN or RLN-only > CLN + RLN	5
Tang (2014) (32)	R	749	7^th^ Edition Stage I–IVB	RLN	–	–	–	83.9%	–	Better prognosis: RLN-only > Other N1 disease; No difference in unilateral vs. bilateral RLN	5
Huang L, Zhang Y, Liu Y (2019) (33)	R	1225	8^th^ Edition Stage I–IVB	RLN Bilateral vs. non-bilateral	1.628	1.178–2.250	0.003	5-year OS: Bilateral: 76.58% vs. Non-bilateral: 88.97%	<0.001	Upgrading bilateral RLN metastasis from N1 to N2	6
**(B) Parotid LN (PLN)**											
Xu (2017) (34)	R	1616	7^th^ Edition Stage I–IVB	PLN	–	–	–	–	0.001	PLN similar prognosis of N3	6
Zhang (2019) (35)	R	10126	8^th^ Edition Stage I–IVB	PLN	–	–	–	–	–	PLN similar prognosis as that of N3	5
**(C) Extra-nodal extension (ENE)**											
Ai (2019) (36)	R	546	8^th^ Edition Stage I–IVB	ENE	G1: 0.637 G2: 1.989	0.396–1.023 1.145–3.457	0.062 0.015	5-year OS: 52.4%	–	Grade 2 ENE (muscle/skin/salivary gland) classified as N3	5
Lu (2019) (37)	R	1616	8^th^ Edition Stage II	ENE	–	–	–	7-year OS: 81.9% vs 89.9%	0.05	ENE associated with poorer prognosis	5
**(D) Lymph node necrosis (LNN)**											
Guo (2015) (38)	R	1197	7^th^ Edition Stage I–IVB	Nodal level (RLN) Extracapsular spread Necrosis Laterality Maximal axial diameter	1.295 - - 1.663 1.326	1.047–1.602 - - 1.200–2.304 0.995–1.767	0.017 - - 0.002 0.054	- -	- NS (0.629) NS (0.130) - -	ENE and LNN is not prognostic	6
Lan (2015) (39)	R	1800	7^th^ Edition Stage I–IVB	LNN	2.03	1.50–2.75	<0.001	5-year OS: LNN 78.8% vs. non-LNN 91.8%	<0.01	N1 with LNN = N2 no LNN N2 with LNN = N3	4
Luo Y, Ren J, Zhou P (2016) (40)	R	189	7^th^ Edition Stage III–IVB	LNN	1.754	1.061–2.899	0.028	5-year OS: 75.8% vs. 59.5%	0.033	N1 with LNN = N2 no LNN N2 with LNN = N3	5
Lu L, Wei X, Li YH (2017) (41)	R	252	7^th^ Edition Stage II–IVB	LNN	2.1	1.57–2.64	<0.01	5-year OS: LNN 74.6% vs. non-LNN 89.7%	<0.01	No suggestion	4
Ting (2017) (44)	R	257	7^th^ Edition Stage I–IVB	Cystic lymph node metastasis (CLNM)	5.785	–	<0.001	N/A	–	CLNM can categorize N2 patients into two prognostic groups	5
Feng (2019) (42)	R	616	8^th^ Edition Stage I–IVB	LNN	2.154	1.282–3.620	0.029	5-year OS: LNN 82.9% vs. non-LNN 93.0%	<0.001	N1 with LNN = N2 no LNN N2 with LNN = N3	6
**(E) Simplification of N-classification**											
Kang (2016) (22)	R	492	7^th^ Edition Stage I–IVB	N3a+ N3b vs. N2	New N3 vs. N2: 2.507	1.508–4.169	<0.001	–	–	Merge N3a and N3b into N3	6
Pan (2016) (14)	R	1609	7^th^ Edition Stage I–IVB	Caudal border of cricoid cartilage to differentiate N2 vs. N3, merging of N3a and N3b	New N3: 4.24	2.57–7.00	<0.001	5-year OS: 7^th^ edition N2/N3a/N3b: 75% vs. 75% vs. 69% Proposed 8^th^ edition: N2/N3: 75% vs 70%	<0.001 <0.001	Merge N3a and N3b into N3	5
Liang (2016) (23)	R	752	7^th^ Edition Stage I–IVB	N1 vs. N2 vs. N3	–	–	–	–	–	Merge N1 and N2	4
**(F) Others**											
Jiang (2017) (43)	R	406	7^th^ Edition N1–3M0	Posterior to level V (PLV) LN	3.431	1.088–10.822	0.035	3-year OS: 51.5% vs. 88.4%	–	PLV LN should be defined as a new lymph node segment	3
Kang (2018) (45)	P	492	7^th^ Edition Stage I–IVB	Cervical LN level	Level VIIa: 1.080 Level III: 1.520 Level IVa: 2.124 Level Va: 0.462 Level Vb: 5.302 Level IVb, Vc: 10.491	0.475–2.452 0.934–2.474 1.041–4.335 0.063–3.381 2.057–13.667 2.483–44.318	–	5-year OS: Level II: 82.7% Level III: 75.2% Level IVa: 67.0% Level Va: 92.3% Level Vb: 37.0% Level IVb, Vc: 33.3% Level VIIa:78.9%	<0.001	N1 [RLN or/and unilateral upper cervical (I, II, III, Va, VIIb, VIII, IX, and X regions) LNs], N2 [bilateral upper cervical LN] and N3 (LN in IVa and Vb regions and their lower regions)	6
Zhou (2018) (46)	R	354	8^th^ Edition Stage I–IVB	Number of lymph node regions (LNR)	2–6: 4.59 ≥7: 9.78	1.36–15.49 2.88–33.25	0.039 0.002	5-year OS: 0–1: 97.1% 2–6: 84.9% ≥7: 74.2%	–	New N classification based on LNR: 0–1, 2–6, ≥7	6
**(III) M-classification (n=5)**											
**Study (Author/Year of publication)**	**Study design (P/R)**	**Sample size**	**Prognostic factor**	**HR (95% CI)**	**P value**	**OS probabilities**	**Survival probabilities**	**P value**	**Proposed changes**	**Quality score**
Shen LJ, Wang SY, Xie GF (2015) (49)	R	505	M1a: single lesion to isolated organ (except for the liver) M1b: single lesion to the liver, or multiple lesions in other organs M1c: multiple lesions in the liver	M1b vs. M1a: 1.69 (1.16–2.48) M1c vs. M1a: 2.64 (1.75–3.98)	0.007 <0.001	3-year OS	M1a: 62.1% M1b: 36.1% M1c: 17.9%	0.001	Recategorization of M stage: M1a: single lesion to isolated organ (except for the liver) M1b: single lesion to the liver, or multiple lesions in other organs M1c: multiple lesions in the liver	4	
Shen (2016) (48)	R	1172	M1a, a single lesion in a single organ or location M1b, multiple lesions in a single organ or location; and M1c, metastases in multiple locations	M1b vs. M1a: 2.28 (1.71–3.05) M1c vs. M1a: 3.65 (2.75–4.85)	–	–	–	–	Subdivision of the M1 stage: M1a, a single lesion in a single organ or location M1b, multiple lesions in a single organ or location; and M1c, metastases in multiple locations	5	
Jiang (2016) (50)	R	347	To use ten-signature classifier * as a classifier for M1a and M1b.	3.45 (2.59–4.60)	<0.001	2-year OS	71.4% vs 18.8%	0.001	To use ten-signature classifier * as a classifier for M1a and M1b.	5	
Tian (2016) (51)	R	263	M1a: 5 single-organ metastases or 1–5 lesions M1b: 5 multiorgan metastases or >6 lesions	N/A	N/A	5-year OS	M1a: 38.7% vs. M1b: 7.0%	<0.001	M1a: oligo metastasis without liver involvement M1b, multiple metastases without liver involvement M1c, liver involvement irrespective of metastatic lesions.	5	
Zou (2017) (52)	R	462 (training set) 272, 243 (internal and external validation set)	M1a: oligometastasis without liver involvement M1b, multiple metastases without liver involvement M1c, liver involvement irrespective of metastatic lesions	M1b vs. M1a: 1.63 (1.17–2.26) M1c vs. M1a: 2.96 (2.14–4.10)	0.004 <0.001	3-year OS	M1a: 54.5–72.8%, M1b: 34.3–41.6%, M1c: 2.6–23.6%	<0.001	M1a: 5 single-organ metastases or 1–5 lesions M1b: 5 multiorgan metastases or >6 lesions	4	

* Ten-signature classifier: oligometastases, extra-regional LN metastases, N-stage, EB-VCA IgA, neutrophil count, platelet count, hemoglobin, glutamic-pyruvic transaminase, glutamyl transpeptidase, and monocyte count

Study design: prospective (P)/retrospective (R); HR, hazard ratio; CI, confidence interval; OS, overall survival.

Among the 64 studies on non-TNM factors, 18 proposed parameters were identified. Prognostic factors with consistent support from multiple studies included EBV-DNA level (**Table 3**), primary gross tumor volume (GTV) (63, 72–74, 76, 78–81), nodal GTV (**Table 4**) (74, 75, 77, 81), neutrophil-lymphocyte ratio (NLR) (**Table 5**) (83, 85, 91, 92, 97), C-reactive protein (CRP)/albumin ratio (83, 89, 98), anemia (84, 87, 96), platelet count (82, 86), lactate dehydrogenase (LDH) (88, 95), and SUV_max_ of the primary tumor (99–101, 103, 108, 111, 113) and total lesion glycolysis (TLG) (**Table 6**) (104, 111).

**Table 3:** Association of overall survival with the pretreatment EBV DNA level (n=16).

Study (Author/Year of publication)	Study design(P/R) ^a^	Sample size	Cutoff value ^b^	HR (high vs. low)	95% CI	*P* value	Survival probabilities	*P* value	Quality score
Chen M, Yin L, Wu J (2015) (53)	P	165	Positive vs negative	–	–	–	2-year OS: Negative EBV 100% vs. positive EBV 94%	1.000	5
Tang (2015) (54)	P/R	6337	4000	8.44	(6.15–11.57)	<0.001	–	–	4
Yang (2015) (55)	R	1168	3760	1.41	(1.06–1.88)	0.017	–	–	4
Zhao (2015) (56)	R	637	1500	1.83	(0.79–4.24)	0.161	–	–	4
Chen (2016) (57)	R	404	4000	3.75	(1.701–8.284)	<0.001	3-year OS: 85% vs. 98%	<0.001	4
Lv (2016) (59)	R	1501	4000	1.97	(1.42 – 2.75)	<0.001	5-year OS: 81% vs. 91%	<0.001	6
Peng H, Chen L, Zhang Y (2016) (117)	R	1106	Positive vs negative	1.83	(1.08–3.11)	0.026	4-year OS: 86% vs. 94%	<0.001	3
Peng H, Guo R, Chen L (2016) (60)	R	584	2010	4.581	(1.58–13.26)	0.005	3-year OS: 92.3% vs. 98.9%	<0.001	3
Zhang (2016) (57)	R	1467	4000	3.44	(2.32–5.09)	<0.001	5-year OS: 83% vs. 95%	<0.001	6
Jin YN, Yao JJ, Zhang F (2017) (61)	R	1036	1500	1.65	(1.10–2.47)	N/A	5-year OS: 79% vs. 87%	0.002	6
Du (2019) (66)	R	607	4000	2.16	(1.25–3.71)	0.005	5-year OS: 85% vs. 97%	<0.001	4
Guo (2019) (67)	R	979	N/A	1.29	(1.13–1.48)	0.001	–	–	4
Huang CL, Sun ZQ, Guo R (2019) (68)	R	949	7000	1.86	(0.77–4.53)	0.171	3-year OS: 95% vs. 89%	0.138	5
Sun XS, Chen WH, Liu SL (2019) (70)	R	2742	1460	3.58	(2.50–5.13)	<0.001	–	–	4
Sun XS, Liang YJ, Liu SL (2019) (69)	R	226	25000	1.91	(1.23–2.96)	0.004	–	–	3
Sun XS, Liu LT, Liu SL (2019) (69)	R	502	Detectable vs undetectable	–	–	–	3-year OS: 34% vs. 69%	<0.001	4

(deleted)^a^Study design: prospective (P)/retrospective (R). ^b^ Cutoff values: pretreatment EBV DNA level (copies/mL).

HR, hazard ratio; CI, confidence interval; OS, overall survival.

N/A, Not available.

**Table 4:** Association of overall survival with tumor volume (n=11).

Study (Author/Year of publication)	Study design(P/R)^a^	Sample size	Primary (P) or nodal (N) cutoff volume (cm^3^)	HR(high vs. low)	95% CI	*P* value	Survival probabilities ^b^	*P* value	Quality score
Tian (2015) (72)	R	229	38 (P)	–	–	–	5-year OS: 15.2% vs. 48.7%	<0.01	4
He (2016) (73)	R	358	46.4 (P)	2.46	1.48–4.10	0.001	3-year OS: 75.5% vs. 90.5%	<0.001	5
Lu (2016) (74)	P	180	20 (P)	–	–	–	5-year OS: 70.6% vs. 95.1%	0.001	4
Qin (2016) (76)	P	249	33 (P)	1.01 (per cm^3^)	1.003–1.018	0.04	5-year OS: 70.5% vs. 86.1%	0.006	3
Luo Y, Gao Y, Yang G (2016) (75)	R	110	14.1 (N)	1.875	1.001–3.512	0.050	5-year OS: 53.0% vs. 75.6%	0.028	4
Chen (2017) (77)	R	1230	7.2/35.7 (N)	1.72 (7.2–35.7 vs. ≤7.2) / 3.41(>35.7 vs. ≤7.2)	1.09–2.69 1.93–6.04	0.019 <0.001	5-year OS: ≤7.2: 90.2% vs. 7.2–35.7: 88.2% vs. >35.7: 62.3%	<0.001	6
Liang (2017) (79)	R	455	28 (P)	3.231	1.776–5.878	<0.001	4-year OS: 75.4% vs. 95.1%	<0.001	4
Liu T, Lv J, Qin Y (2017) (81)	P	143	43.5 (P) 15.0 (N)	7.81 3.55	1.79–33.3 1.33–9.43	0.006 0.011	5-year OS: 68% vs. 97% 5-year OS: 74% vs. 91%	<0.001 <0.001	5
Zhang (2017) (78)	R	393	23 (P)	2.05	1.11–3.80	0.022	–	–	3
Chen (2018) (63)	P	385	30 (P)	–	–	–	3-year PFS: 89% vs. 96%	0.008	4
Jeong (2018) (80)	R	133	33 (P)	2.93	1.16–7.42	0.013	5-year OS: 67% vs. 87%	0.021	5

^a^Study design: prospective (P)/retrospective (R). ^b^Survival outcomes: OS, overall survival; PFS, progression-free survival. HR, hazard ratio; CI, confidence interval

**Table 5:** Association of overall survival with neutrophil-lymphocyte ratio (NLR) (n=4).

Study (Author/Year of publication)	Study design(P/R)^a^	Sample size	Prognostic factors	Cut off point	HR(high vs. low)	95% CI	*P* value	Survival probabilities	*P* value	Quality score
Lu AY, Li HF, Zheng YM (2019) (85)	R	140	NLR	≥2.28	2.383	1.041–5.457	0.040	5-year OS: ≥2.28: 70.3% <2.28: 87.8%	0.010	4
Ye (2018) (91)	R	427	NLR	≥2.32	1.699	1.005–2.873	0.048	5-year OS: ≥2.32: 81.8% <2.32: 90.0%	0.015	4
Akcay (2019) (92)	R	62	NLR	≥3	1.19	1.04–2.35	0.002	NA	NA	5
Yao (2019) (97)	R	1550	NLR	>2.50	1.72	1.31–2.24	<0.001	5-year OS: >2.50: 82.5% ≤2.50: 90.3%	<0.0001	6

^a^Study design: prospective (P)/retrospective (R).

HR, hazard ratio; CI, confidence interval; OS, overall survival.

N/A, Not available.

**Table 6:** Association of overall survival with PET parameters (n=10).

Study(Author/Year of publication)	Study design(P/R)^a^	Sample size	Nature of PET parameters	Cutoff value	HR (high vs. low)	95% CI	*P* value	Survival probabilities(overall survival)	*P* value	Quality score
Yoon (2014) (99)	R	53	SUV_max_-T MTV-T_2.5_ MTV-T_3.0_	≥8.9 ≥31.45 cm^3^ ≥23.01 cm^3^	1.08 5.03 5.03	0.25–7.71 1.04–24.24 1.04–24.24	0.74 0.029 0.029	–	–	3
Zaghloul (2014) (100)	R	70	SUV_max_-T	≥8.0	–	–	0.034 (U)	–	–	3
Hsieh (2015) (101)	R	174	SUV_max_-T	≥8.35	3.91	1.45–10.53	0.007	5-year OS: 69.2% vs. 93.4%	0.001	4
Shen T, Tang LQ, Luo DH (2015) (102)	R	194 (107: LR; 87: DM)	SUV_max_-T or SUV_max_-M	≥8.65 (LR) ≥13.55 (DM)	4.882 2.415	1.06–22.59 0.96–6.10	0.042 0.062	–	–	4
Xiao W, Xu A, Han F (2015) (103)	R	179	SUV_max_-T	≥10.22	–	–	–	5-year OS: 66.6% vs. 87.6%	<0.001	5
Yoon (2016) (104)	R	97	TLG	322.7	0.29	0.11–0.79	0.02	5-year OS: 54.0% vs. 85.7%	0.003	6
Lee (2017) (108)	R	53	SUV_max_-T SUV_max_-N	≥13.2 ≥13.4	- 7.799	- 1.51–40.40	- 0.014	- 3-year OS: 33.1% vs. 55.5%	- 0.003	5
Liu F, Xi XP, Wang H (2017) (109)	R	213	PET-CT-guided dose-painting IMRT vs CT-based IMRT	–	0.425	0.18–1.009	0.052	3-year OS: 82.6% vs. 91.8%	0.049	5
Alessi (2019) (111)	R	160	SUV_max_-T SUV_mean_-T TLG	18.8 9.5 382.2	1.07 1.07 1.003	- - -	0.01 0.01 0.01	–	–	3
Sun XS, Liang YJ, Liu SL (2019) (113)	R	253	SUV_max_-T, SUV_max_-N SUV_max_-M	17.0 12.7 6.9	- 1.40 1.72	- 0.95–2.06 1.13–2.78	- 0.087 0.012	3-year OS: SUV_max_-T: >17 vs. ≤17: 47.7% vs. 57.3% 3-year OS: SUV_max_-N: >12.7 vs. ≤12.7: 46.5% vs. 65.1% 3-year OS: SUV_max_-M: >6.9 vs. ≤6.9: 49.2% vs. 65.4%	SUV_max_-T: 0.48 SUV_max_-N: 0.005 SUV_max_-M: 0.005	5

^a^Study design: prospective (P)/retrospective (R).

HR, hazard ratio; CI, confidence interval; SUV_max_, maximum standardized uptake value; SUV_mean_, mean standardized uptake value; MTV, metabolic tumor volume; TLG, total lesion glycolysis; T, primary tumor; N, lymph node; M, metastasis; LR, local recurrence.

## Discussion

To our understanding, this systematic review that evaluated the prognostic factors for NPC patients in 108 articles published from 2013 to 2019 is the most comprehensive review on this topic. The TNM 8^th^ Edition, based entirely on the anatomical tumor extent, is the most widely used prognostic tool for NPC and remains the most robust factor for guiding treatment decisions, evaluating treatment results, and comparing outcomes between institutions worldwide. However, continuous improvement is necessary in view of the advances in investigations and treatments. Furthermore, refinement of prognostic tools by the incorporation of novel proposals based on functional imaging, plasma biomarkers, and molecular tumor characteristics is desirable in the current era of personalized oncology. For tumors with disease sites such as the prostate, breast, and skin (i.e., melanoma), non-anatomical factors have been successfully incorporated while still maintaining essential anatomical information. For NPC, considerable progress on both anatomical and non-anatomical prognostic factors have been made since the publication of the TNM 8^th^ Edition. This systematic review reviewed the latest evidence to facilitate the formulation of a comprehensive proposal for designing the upcoming TNM 9^th^ Edition.

### T-Classification

A major change in the TNM 8^th^ Edition was the replacement of the ambiguous terms IF/masseter space involvement with a clear specification of extensive soft tissue infiltration beyond the lateral surface of LP as T4, and the downstaging of MP/LP/PM to T2. This change was supported by two studies (10, 13, 14). However, five studies showed that MP and/or LP involvement was associated with a worse prognosis than T2 and should be upstaged; suggestions included categorizing MP as T3 and LP as T4 disease (n = 1) (16), MP as T2 and LP as T4 (n = 3) (13, 15, 30), and both MP and LP as T3 disease (n = 1) (10). Thus, further validation of the prognostic significance of MP/LP is recommended.

Three studies, comprising a total of 1,348 patients, showed that PNS involvement should be upstaged from current T3 to T4 disease given its poorer outcomes (5-year OS rate of 53.7–83.7%) (16, 19, 20). Of note, Zhang et al. reported worse prognoses among patients with ethmoid sinus or maxillary sinus involvement as T4 disease, but better prognosis in those with sphenoid sinus invasion alone as T3 disease (18); further studies on the relapse risks of various PNS are warranted.

The widespread use of magnetic resonance imaging (MRI) has improved the accuracy of detection of the extent of involvement of the skull base and of intracranial extension. With better disease characterization, Li et al. proposed the subdivision of skull base involvement into T3-slight (pterygoid process and/or base of the pterygoid bone only) and T3-severe (others) (24); similarly, Cao et al. suggested the subdivision of T4 into T4a (without intracranial extension) and T4b (with intracranial extension) based on the presence of intracranial extension (29). Further studies are needed to validate these findings.

With the technological advances in both diagnostics and treatment, the differences in survival and local control in the T-category has diminished. Eight of the included studies proposed the simplification of the T-category (6, 21–27); these included three studies that suggested the merging of T1 and T2 disease (21, 23, 24), one suggested combining of T1, T2, and T3 disease (22), and one proposed a merging of T2 and T3 (27). Other studies proposed simplification of the definition of T-classification, refinement of T2–T4 disease, and reclassification as T1 and T2 only (6, 25, 26).

**Level of Evidence:**

**Strong:** PNS involvement (T4 disease)

**Moderate:** Nil

**Inconclusive:** MP (upstaged to T3/4), LP (upstaged to T3/4), and merging of T-classification (T1–T2, T1, T2, and T3, or T2–T3)

**Limited:** Skull base (T3 slight *vs.* T3 severe) and intracranial extension (T4a *vs.* T4b).

### N-Classification

Despite the rarity of PLN metastasis (0.4–2.8%), consistent findings were noted on its adverse prognostic outcome, which was similar to those with N3 disease, as demonstrated in two studies that included a total of 11,742 patients. Both reports recommended PLN involvement as the criteria for N3 classification (34, 35). Also, suspicion of PLN metastasis, especially in patients with advanced nodal diseases, should be raised on pretreatment imaging, and biopsy is indicated in the suspected case.

Furthermore, in five studies, there was consensus that LNN was an adverse prognostic factor (hazard ratio [HR]: 1.75–5.79) (38–42). In the largest study by Lan et al., patients with LNN had worse OS and DMFS (OS, 78.8 *vs.* 91.8%; DMFS, 78.4 *vs.* 91.6%, both *p* < 0.001); the authors proposed that patients with LNN should be upstaged in their respective N-category (39).

In addition to the proposals identified in the current literature search, extra-nodal extension (ENE) was recently advocated as a new criterion for N3-classification in the TNM 8^th^ Edition for other head and neck cancers, but not for NPC. Specifically, Ai et al. proposed the categorization, as N3 disease, of ENE with infiltration into the adjacent muscle/skin/salivary gland (36). Lu et al. showed that ENE was a poor prognostic factor for NPC and proposed to categorize ENE as G0: lymph nodes without ENE; G1: tumor infiltration beyond the individual nodal capsule(s) into the surrounding fat plane; G2: coalescent nodal mass with unequivocal evidence of ENE; G3: tumor infiltration beyond the nodal capsule into adjacent structures (37). Only G2/G3 ENE, but not G1, was independently prognostic of death; the authors hence proposed a refined N-classification: New-N1: N1/N2 without G2-/G3-ENE; New-N2: N1 with G2-ENE; New-N3: N2 with G2-ENE, N1/N2 with G3-rENE, or N3. On the contrary, Guo et al. suggested that ENE was not a poor prognostic factor; but the definition of ENE was not mentioned in their study (38).

The current TNM 8^th^ Edition categorizes retropharyngeal lymph node involvement (≤6 cm) as N1 disease, regardless of its unilateral or bilateral involvement. Tang et al. supported the current classification (32), but Study by Huang et al. on 1,225 patients (33) suggested upstaging bilateral retropharyngeal lymph node involvement as N2 disease, as they have worse 5-year OS (89.4 *vs.* 82.6%) and DMFS (91.5 *vs.* 82.9%).

Furthermore, four studies proposed the simplification of the N-classification and supported the current N3 disease with merging of the previous N3a and N3b (14, 23, 45, 47). Other studies on PLV LN, cervical LN level, and the number of LN regions had limited evidence (22, 43, 44, 46).

**Level of Evidence:**

**Strong:** PLN (N3 disease), LNN (Upstaging of N-classification)

**Moderate:** ENE (Upstaging of N-classification or N3)

**Inconclusive:** RLN involvement

**Limited:** PLV LN, cervical LN level, number of LN regions, merging of N2 and N3

### M-Classification

Several suggestions have been made on the subcategorization of *de novo* oligo-metastatic disease based on the number of metastatic lesions and the site(s) of involvement (48–52). However, given the diversity of definition and management of patients with oligo-metastasis, no conclusive recommendation could be made. Most studies have shown that the number of metastatic lesions and the number of organ involvements were independent poor prognostic factors. Furthermore, both Shen et al. and Zou et al. reported that single (or oligo-) metastatic lesions without liver involvement had better prognoses compared with lesions with liver involvement (49, 52). In a multicenter study of 977 patients that was reported by Zou et al., liver metastases represented a worse prognostic factor regardless of the number of metastatic lesions with a 3-year OS rate of 34.3–72.8% *vs.* 22.6–23.6% (52).

**Level of Evidence:**

**Inconclusive:** Subclassification of M-category

### Plasma EBV-DNA Level

The measurement of EBV-DNA concentration is widely used in diagnosis, prognostication, treatment monitoring, and the surveillance of recurrence. In concordance with previous meta-analyses (115–117), we found that the pretreatment plasma EBV-DNA level was a prognostic factor; the risk of mortality, local failure, and metastases was 1.3- to 8.4-fold, 1.1- to 3.1-fold, and 1.4- to 8.1-fold higher, respectively, for patients with high EBV-DNA levels compared to patients with low EBV-DNA levels (53–71, 114).

Several studies have highlighted the important role of EBV-DNA to refine the prognosis of patients with similar TNM stage groups. In a study of 385 patients with Stage II (TNM 7^th^ edition) disease, the 3-year PFS, LRFS, and DMFS rates for the detectable and undetectable EBV-DNA groups were 89.1 *vs.* 96.4%, 94.3 *vs.* 98.2%, and 94.2 *vs.* 98.6%, respectively (*p* = 0.005, 0.039, and 0.017, respectively) (63). For locally advanced disease, Zhang et al. revealed that patients with stage II–III (TNM 7^th^ Edition) and a high EBV-DNA level had worse survival than those with stage IVa–b and a low EBV-DNA level (5-year OS: 82.7 *vs.* 92.9%, PFS: 70.7 *vs.* 89%) (57). Similarly, Jin et al. showed that the prognosis of patients with stage IVa–b (TNM 7^th^ Edition) and low EBV-DNA level was similar to that of patients with Stage III disease and high EBV-DNA level (61).

Furthermore, two studies demonstrated that recursive partitioning analysis (RPA), which integrated stage groups and the plasma EBV-DNA level, had better survival predictive ability compared to the TNM 8^th^ Edition (67, 71). Guo et al. proposed the following RPA classes: Stage RI (T1N0), RIIA (T2–T3N0 or T1–T3N1, EBV-DNA ≤2,000 copies/ml), Stage RIIB (T2–T3N0 or T1–T3N1, EBV-DNA >2,000 copies/ml; T1–T3N2, EBV-DNA ≤2,000 copies/ml), Stage RIII (T1–T3N2, EBV-DNA >2,000 copies/ml; T4N0–N2), and Stage RIVA (any T and N3) (67). The 5-year PFS rate was 100, 87.9, 76.7, 68.7, and 50.4% for the proposed stages RI, RIIA, RIIB, RIII, and RIV, respectively (*p* < 0.001). In a similar study by Lee VH et al., RPA derived four new stages: RPA-I (T1–T4, N0–N2, and EBV-DNA <500 copies/ml), RPA-II (T1–T4, N0–N2, and EBV-DNA ≥500 copies/ml), RPA-III (T1–T2 and N3), and RPA-IVA (T3–T4 and N3) (71).

The EBV-DNA concentration could provide biological information of tumors beyond the anatomical factors and thereby improve the prognostic performance of the staging system. Nonetheless, the heterogeneity of cutoff values has hindered the wide application of EBV-DNA in NPC staging. The EBV-DNA cutoff values varied markedly among our included studies (1,500–25,000 copies/ml), with 4,000 copies/ml being the most frequently used cutoff value (54, 57, 59, 66). Plasma EBV-DNA is a laboratory-developed test with heterogeneity based on different DNA extraction, purification, and stabilization methods; different instruments used; different primers and probes that target a different part of the EBV genome; and different quantification controls (118). An earlier study showed that different PCR assays using primer/probe sets for latent membrane protein-2 (LMP-2) and BamHI-W might yield slightly different plasma EBV-DNA concentrations from that in the same sample (119). Also, the low sensitivity of EBV-DNA assays in patients with low-volume NPC is another concern (120). Thus, further international efforts are encouraged to harmonize the assay and validate it in large prospective cohorts to ensure that plasma EBV-DNA can unleash its full potential and be incorporated into the staging system.

**Level of Evidence:**

**Strong:** Pretreatment EBV-DNA level

### Tumor Volume

There were 12 studies with 8,403 patients in the current systematic review that evaluated the significance of tumor volumes (GTV-P and/or GTV-N). Seven papers focused on the primary tumor volume (GTV-P; n = 7) (63, 72, 73, 76, 78–80), two on the nodal tumor volume (GTV-N; n = 2) (75, 77), and two on the total tumor volume including primary and node (GTV-P and GTV-N; n = 2) (74, 81). One study did not include a cutoff for GTV-N and GTV-P (65).

The findings suggested that large GTV-P was an independent predictor of OS (HR 1.56–3.23) (63, 72–74, 76, 78–80), DMFS (HR 1.01–3.23) (63, 65, 77–81), and LRFS (HR 1.01–2.79) (63, 73, 76, 78–81). Similarly, large GTV-N was an adverse prognostic factor for OS (HR 1.56–3.41) (75, 77) and DMFS (HR 2.72–6.33) (75, 81). However, the proposed cutoff values varied widely among the studies included in this review (**Table 4**): GTV-P ranged from 20 to 50 ml (median 33 ml), and GTV-N ranged from 7.2 to 35.7 ml (median 15 ml).

The current T- and N-classifications of the staging system are primarily based on the extent of tumor invasion and the maximum diameter of the LN, respectively. Tumor volume might correlate better with the number of clonogenic tumor cells, leading to a more accurate prediction of the chance of cure (121). Volumetric stratification has been demonstrated to improve the prognostic ability of the TNM staging system. Jeong et al. divided stage II–IV (TNM 8^th^ Edition) into the volume subgroup and found that the 5-year OS was significantly better in participants with GTV-P ≤33 ml compared to those with GTV-P >33 ml (87.3 *vs.* 66.7%) (80); Chen et al. showed that among 385 TNM-8^th^ Edition classified Stage II patients, those with a total GTV <30 cm^3^ was associated with a better prognosis than those with a total GTV ≥30 cm^3^ (63).

Despite the growing body of evidence, tumor volume is yet to be used for cancer staging in routine clinical practice for several reasons. Firstly, there are significant intra- and inter-observer variations in volume delineation. Secondly, the malignant tumor often grows into irregular shapes, and accurate measurement of tumor volume is hard to achieve with conventional imaging. Furthermore, the cutoff value of the tumor volume is difficult to define due to the differences in assessment software, measurement timing, and methods of statistical analysis (122, 123). Future efforts are needed to overcome these challenges before tumor volume can be used as a widely applied prognostic marker.

**Level of Evidence:**

**Strong:** Primary GTV volume and nodal GTV volume

### Blood Inflammatory/Hematological Markers

In the 18 studies that were included, nine inflammatory/hematological markers were evaluated: the most frequently proposed marker (n = 5) is NLR (83, 85, 91, 92, 97), followed by anemia (n = 3) (84, 87, 96), LDH (n = 2) (88, 95), platelet count (n = 2) (82, 86), and the CRP/albumin ratio (n = 3) (83, 89, 98). Other proposals with limited supporting evidence included high-sensitivity CRP (hs-CRP; n = 1) (54), platelet distribution width (PDW) (86), prognostic nutrition index (PNI) and albumin/globulin ratio (AGR) (n = 1) (93), D-dimer (n = 1) (94), and tumor-infiltrating lymphocytes (TIL; n = 1) (90).

The results of 2,225 NPC patients in five studies showed that elevated pretreatment NLR was consistently associated with worse OS (HR 1.19–2.38), DMFS (HR 1.45), and LRFS (HR 1.35) (**Supplementary Table 5**) (83, 85, 91, 92, 97). Evidence suggested that proinflammatory tumor microenvironments are closely related to cancer development and progression. Lymphocytes are immune cells that exhibit an antitumor function, while neutrophils are inflammatory cells that influence the cytotoxic activity of the immune system. Therefore, an increased NLR, with an elevated neutrophil count and/or reduced lymphocyte count, is a biomarker that reflects an imbalance in pro- and antitumor activities in the host’s immune system. Various cutoff values of NLR have been suggested (range 2.28–3.00, median 2.32), and the analysis suggested that NLR was a reliable prognostic marker regardless of the cutoff value (124).

Other hematological markers such as hemoglobin, platelet count, LDH, and CRP have the advantages of easy accessibility, inexpensive measurement, and high reproducibility and therefore possess a promising potential for integration into the international prognostic system. In particular, the significance of LDH and CRP have long been recognized (125–127), and these parameters had been incorporated in various recently published prognostic nomograms of NPC (128–130). Accordingly, further validations of these findings are encouraged.

**Level of Evidence:**

**Strong:** NLR, CRP/albumin ratio, anemia, PDW and platelet count, and LDH

**Limited:** Hs-CRP, PNI and AGR, D-dimer, and TIL

### FDG-PET Parameters

Among the 15 studies on FDG-PET included in the current review, most evaluated the maximum SUV (SUV_max_), either alone (n = 11) (100–103, 105–108, 110, 112, 113); some also proposed other metabolic parameters, such as metabolic tumor volume (MTV; n = 1) (99) or TLG (n = 2) (104, 111) (**Supplementary Table 6**). A single study further evaluated the difference in prognosis between PET-CT-guided dose-painting intensity-modulated radiation therapy (IMRT) and CT-based IMRT (109).

Four studies consistently showed that the high SUV_max_ of the primary tumor was associated with poor OS (HR 1.07–4.88) (99, 101, 102, 111); however, conflicting results were shown with regard to the high SUV_max_ of nodal and metastatic disease (102, 113). High TLG was associated with inferior OS in two studies, and MTV was a poor prognostic factor in one study (**Supplementary Table 6**) (99, 104, 111). Therefore, we recommend further validation of the role of the high SUV_max_ of the primary tumor and high TLG.

The metabolic information of FDG-PET could predict tumor aggressiveness and be correlated with patient survival (131). The majority of FDG-PET studies evaluated the prognostic role of the SUV_max_ of the tumor mass; however, the SUV_max_ was limited by representing only the maximum uptake within the volume of interest (VOI) instead of within the entire mass. Emerging metabolic parameters such as TLG and MTV have been proposed to overcome these limitations: MTV is measured by contouring margins defined by thresholds, whereas TLG is calculated by multiplying the MTV by the mean SUV. Additional studies are encouraged to define the prognostic role of the abovementioned factor. However, the diverse range of cutoff values of these PET parameters used in different studies are attributable to several reasons. First, variables such as tumor delineation and definition of VOI may affect the MTV and TLG values; second, the cutoff values are established by the statistical parameters of each institution without cross-validation. Based on the evidence in the current literature, we cannot recommend a concrete cutoff value for further validation as the wide range of values has limited its reproducibility and global applicability.

**Level of Evidence:**

**Strong:** High SUV_max_ of the primary tumor and TLG

**Limited:** MTV

**Inconclusive:** High SUV_max_ of nodal disease and SUV_max_ of metastatic disease

### Limitations

The limitations of this research merit discussion. Firstly, despite the exclusion of poor-quality studies, most of the included studies had a retrospective observational design, which is prone to biases. Secondly, the majority of the included studies that evaluated the non-anatomical markers used dichotomous variables to determine the prognostic value. The cutoff value of parameters varied among different studies, as it was calculated statistically in each study to achieve the most significant prognostic effect; therefore, the generalizability of the findings is uncertain. Thirdly, due to the heterogeneity of study designs, study populations, measurement techniques, and cutoff values, we were unable to perform a meta-analysis to estimate a pooled value reliably. Also, some of the studies of plasma EBV-DNA in early years were not included in the present analysis; however, our conclusion remains consistent with the previous findings (115–117). Lastly, some of the novel markers, such as radiomics, micro-RNA, circulating tumor cells, and genetic signatures, were not included in this review due to their limited global applicability at present.

### Summary Remarks

This systematic review has identified a comprehensive list of prognostic factors and suggestions that could contribute toward more accurate risk stratification for designing personalized treatment for NPC. Further studies for the validation of these factors are needed to confirm reproducibility and define the optimal cutoff criterion, to formulate the recommendations for designing the upcoming 9^th^ Edition of the TNM staging system.

## Data Availability Statement

The original contributions presented in the study are included in the article/**Supplementary Material**. Further inquiries can be directed to the corresponding author.

## Author Contributions

WN, JP, and AL: conception and design. CC, QG, TM, ZX, HC, and JL: collection and assembly of data. CC, WN, HC, JP, and AL: data analysis and interpretation. CC, WN, and AL: manuscript writing. All authors contributed to the article and approved the submitted version.

## Conflict of Interest

CC: Consulting or Advisory Role: AstraZeneca, Eiasi; Research funding: AstraZeneca, Merck Kgga.

The remaining authors declare that the research was conducted in the absence of any commercial or financial relationships that could be construed as a potential conflict of interest.

## Publisher’s Note

All claims expressed in this article are solely those of the authors and do not necessarily represent those of their affiliated organizations, or those of the publisher, the editors and the reviewers. Any product that may be evaluated in this article, or claim that may be made by its manufacturer, is not guaranteed or endorsed by the publisher.
