# Supplementary material for: Prognostic Factors for Overall Survival in Nasopharyngeal Cancer and Implication for TNM Staging by UICC: A Systematic Review of the Literature
Source: Front Oncol. 2021 Sep 2;11:703995. doi: 10.3389/fonc.2021.703995 (PMC8445029; doi:10.3389/fonc.2021.703995)
Supplement: Supplementary file 1 [file Table_1.docx]

Supplementary Material

# Supplementary Table 1. Search Strategy

**Search date: September 13, 2019**

| PubMed |  |
| --- | --- |
| Criteria | Search term |
| Diseases | ((nasopharyn*[Title] AND (carcinoma[Title] OR cancer[Title] OR neoplasm*[Title])) OR (NPC[Title] AND nasopharyn*[Text])) |
| Theme | (stag*[Title/Abstract] OR TNM[Title/Abstract] OR prognos*[Title/Abstract]) |
| Language | ("Chinese"[Language] OR "English"[Language]) |
| Publication date | ("2014/01/01"[Date - Publication] : "3000"[Date - Publication]) |
| Items returned | 1,758 |
|  |  |
| EMBASE |  |
| Criteria | Search term |
| Diseases | (nasopharyn* AND (carcinoma OR cancer OR neoplasm*)).ti. OR (NPC.ti. AND nasopharyn*.tw.) |
| Theme | (stag* or TNM or prognos*).ab,ti. |
| Language | (Chinese OR english).lg. |
| Publication date | ("2014" OR "2015" OR "2016" OR "2017" OR "2018" OR "2019").yr. |
| Items returned | 1,769 |
|  |  |
| Scopus |  |
| Criteria | Search term |
| Diseases | ( TITLE ( nasopharyn* AND (carcinoma OR cancer OR neoplasm*)) OR (TITLE(NPC) AND nasopharynx*)) |
| Theme | TITLE-ABS-KEY ( stag* OR TNM OR prognos* ) |
| Language | LANGUAGE ("English" OR "Chinese") |
| Publication date | PUBYEAR > 2013 AND PUBYEAR < 2020 |
| Items returned | 2,068 |
|  |  |
| Overall items returned | 5,595 |

# Supplementary Figure 1. Study inclusion flowchart

**
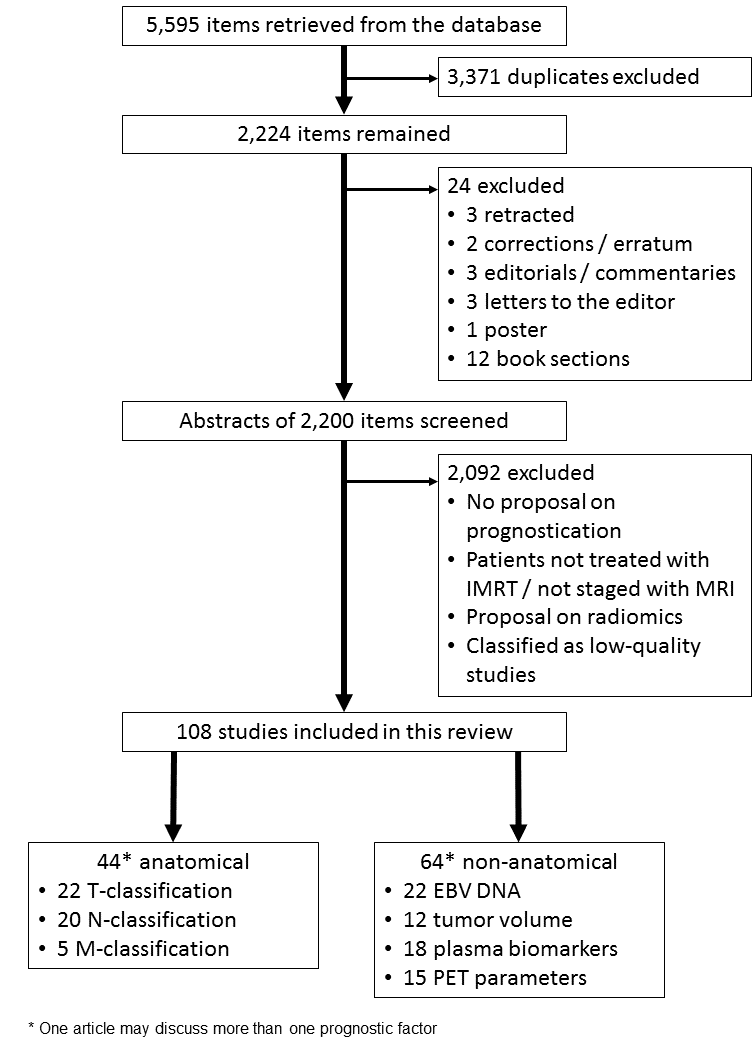
**

# Supplementary Table 2. Characteristics of studies of T-, N-, and M-classification prognostic factors and survival outcomes (n=44)

| **(I) T-classification (n=22)** | | | | | | | | | | | | | | | | | | | | | | | | | | | | | | | | | | | |
| --- | --- | --- | --- | --- | --- | --- | --- | --- | --- | --- | --- | --- | --- | --- | --- | --- | --- | --- | --- | --- | --- | --- | --- | --- | --- | --- | --- | --- | --- | --- | --- | --- | --- | --- | --- |
| Study  (Author/Year of publication) | Study design  (P/R) ^a^ | | | Sample size | | | IMRT  (Y/N) | | | MRI staging  (Y/N) | | | TNM/UICC staging | | | Prognostic factor | Survival outcomes ^b^ | | | | | | | | | | Quality score ^c^ | | | | | | | Changes proposed by authors | |
|  |  | | |  | | |  | | |  | | |  | | |  | OS | PFS | | DMFS | | | LRFS | | |  | | | | | | |  | |  |
| **(A) Medial pterygoid (MP), lateral pterygoid (LP), prevertebral muscle (PM), and infratemporal fossa (IF)** | | | | | | | | | | | | | | | | | | | | | | | | | | | | | | | | | | | |
| Luo (2014)^10^ | R | | | 742 | | | Y | | | Y | | | 7^th^ Edition  Stage III–IVB | | | MP, LP | Y | Y | | Y | | | Y | | | 5 | | | | | | | MP/LP should be staged as T3 | |  |
|  |  | | |  | | |  | | |  | | |  | | | IF | Y | N | | N | | | N | | |  | | | | | | | IF should be staged as T4 | |  |
| Sze (2014)^11^ | R | | | 1104  434 (T3) | | | IMRT (36.9%) | | | MRI (99.9%) | | | 7^th^ Edition  Stage I–IVB | | | MP ± LP | Y | N | | Y | | | Y | | | 5 | | | | | | | MP ± LP should be classified as T2 | |  |
| Zhang (2014)^30^ | R | | | 808 | | | Y | | | Y | | | 7^th^ Edition  Stage I–IVB | | | Masticatory space involvement | Y | N | | Y | | | Y | | | 4 | | | | | | | Medial involvement to be classified as T2; lateral involvement to be classified as T4 | |  |
| Xiao Y, Pan J, Chen Y (2015)^12^ | R | | | 816 | | | IMRT (36%) | | | Y | | | 7^th^ Edition Stage I–IVB | | | MP | Y | N | | Y | | | Y | | | 4 | | | | | | | MP similar to T2 prognosis | |  |
| Zhang (2015)^13^ | R | | | 1504 | | | Y | | | Y | | | AJCC6 | | | Medial pterygoid muscle, lateral pterygoid muscle, posterior carotid space, paraoropharyngeal extension | Y | N | | Y | | | Y | | | 3 | | | | | | | T2: Mild invasion (involvement of medial pterygoid muscle of masticator space or pterygoid, carotid, prevertebral, or retropharyngeal spaces)  T4: extensive invasion (involvement of lateral pterygoid muscle and beyond of masticator space or parotid space). | |  |
| Pan (2016)^14^ | R | | | 1609 | | | Y | | | Y | | | 7^th^ Edition Stage I–IVB | | | MP/LP/PM | Y | N | | N | | | Y | | | 6 | | | | | | | MP/LP/PM as T2 | |  |
| Kang M, Zhou P, Liao X (2017)^15^ | R | | | 608 | | | Y | | | Y | | | 8^th^ Edition Stage I–IVB | | | MP/LP | Y | Y | | Y | | | Y | | | 4 | | | | | | | LP should be graded as T4  MP: T2 | |  |
| Zhou (2017)^16^ | R | | | 358 | | | Y | | | Y | | | 7^th^ Edition  Stage III–IVb | | | MP, LP, PNS, skull base, cavernous sinus, and CN | Y | Y | | Y | | | Y | | | 5 | | | | | | | T3: MP and skull base  T4: LP, PNS, MS beyond LP, Cavernous sinus, and CN | |  |
| **(B) Skull base involvement** | | | | | | | | | | | | | | | | | | | | | | | | | | | | | | | | | | | |
| Li (2019)^17^ | R | | | 1225 | | | Y | | | Y | | | 8^th^ Edition  Stage I–IVB | | | Skull base (T3 slight: pterygoid process and/or base of pterygoid bone vs. T3 severe) | Y | Y | | N | | | N | | | 6 | | | | | | | Patients with **T3 slight** (base of pterygoid bone and pterygoid process) should be T2; **T3 severe** remains as T3 | |  |
| **(C) Paranasal sinus (PNS) involvement** | | | | | | | | | | | | | | | | | | | | | | | | | | | | | | | | | | | |
| Zhang (2016)^18^ | R | | | 1811 | | | Y | | | Y | | | 7^th^ Edition Stage I–IVB | | | PNS | Y | N | | Y | | | N | | | 5 | | | | | | | T3: Sphenoid sinus  T4: Ethmoid and maxillary sinuses | |  |
| Zhou (2017)^16^ | R | | | 358 | | | Y | | | Y | | | 7^th^ Edition  Stage III–IVb | | | MP, LP, PNS, skull base, cavernous sinus, and CN | Y | Y | | Y | | | Y | | | 5 | | | | | | | T3: MP and skull base  T4: LP, PNS, MS beyond LP, Cavernous sinus, and CN | |  |
| Wang Y, Zhao J, Zhao Y (2018)^19^ | R | | | 295 | | | Y | | | Y | | | 8^th^ Edition  T3–4, M0 | | | PNS | Y | Y | | Y | | | Y | | | 3 | | | | | | | PNS to be moved to T4 | |  |
| Cao (2019)^20^ | R | | | 695 | | | Y | | | Y | | | 8^th^ Edition  Stage I–IVb | | | PNS | Y | Y | | Y | | | Y | | | 5 | | | | | | | PNS to be reclassified to T4 | |  |
| **(D) Intra-cranial extension or cranial nerve involvement** | | | | | | | | | | | | | | | | | | | | | | | | | | | | | | | | | | | |
| Zong (2014)^28^ | R | | | 375 | | | Y | | | Y | | | 7^th^ Edition  Stage III–IVB | | | CN involvement | Y | N | | Y | | | Y | | | 4 | | | | | | | MRI-detected CN should not be reclassified into T4 to avoid excessive treatment | |  |
| Cao (2017)^29^ | R | | | 335 | | | Y | | | Y | | | 7^th^ Edition  T4 | | | Intracranial invasion | Y | Y | | Y | | | Y | | | 5 | | | | | | | To subclassify T4: (a) without intracranial invasion, (b) with intracranial invasion | |  |
| **(E) Simplification of T-classification** | | | | | | | | | | | | | | | | | | | | | | | | | | | | | | | | | | | |
| Zong (2015)^21^ | R | | | 1241 | | | Y | | | Y | | | 7^th^ Edition Stage I–IVB | | | T1 vs. T2 vs. T3 | Y | N | | N | | | N | | | 4 | | | | | | | Merge T1–T2 | |  |
| Kang M, Long J, Li G (2017)^22^ | R | | | 492 | | | Y | | | Y | | | 7^th^ Edition Stage I–IVB | | | T1 vs. T2 vs. T3 vs. T4 | Y | N | | Y | | | Y | | | 4 | | | | | | | Merge T1–T3 to T1 and classify T4 as T2 | |  |
| Liang (2016)^23^ | R | | | 752 | | | Y | | | Y | | | 7^th^ Edition  Stage I–IVB | | | T1 vs. T2 vs. T3 vs. T4 | Y | N | | N | | | Y | | | 4 | | | | | | | Merge T1–T2 to T1,  T3 as T2, and T4 as T3 | |  |
| Li (2018)^24^ | R | | | 382 | | | Y | | | Y | | | 7^th^/8^th^ Edition  Stage I–IVB | | | T1 vs. T2 vs. T3 vs. T4 | Y | Y | | Y | | | Y | | | 6 | | | | | | | Merge T1–T2 | |  |
| Yang (2018)^25^ | R | | | 1317 | | | Y | | | Y | | | 7^th^/8^th^ Edition  Stage I–IVB | | | T1 vs. T2 vs. T3 vs. T4 | Y | N | | Y | | | N | | | 6 | | | | | | | Refinement of T2–T4 staging needed as there is no difference in LRFS in T2–T4; and OS difference in T2 and T3 | |  |
| Kang M, Zhou P, Wei T (2017)^26^ | P | | | 492 | | | Y | | | Y | | | 7^th^ Edition Stage I–IVB | | | T1 vs. T2 vs. T3 vs. T4 | Y | N | | Y | | | N | | | 5 | | | | | | | T1 (nasopharynx, nasal cavity, parapharyngeal space, oropharynx, skull base, and MP); T2 (LP, paranasal sinus, infratemporal fossa, orbit, cranial nerves, cavernous sinus and intracalvarium) | |  |
| Pan (2019)^6^ | R | | | 325 | | | Y | | | Y | | | 8^th^ Edition stage I–IVB | | | T1-T4 | N | N | | N | | | N | | | 4 | | | | | | | Simplification of the definition of T1, T2, T3, and T4 | |  |
| Tang (2019)^27^ | R | | | 2191 (training set); 414 (validation set) | | | Y | | | Y | | | 8^th^ Edition  Stage I–IVB | | | T1 vs. T2 vs. T3 vs. T4 | Y | Y | | N | | | Y | | | 6 | | | | | | | Merge T2 and T3 to the proposed T2 (proT2) | |  |
| **(II) N-classification (n=20)** | | | | | | | | | | | | | | | | | | | | | | | | | | | | | | | | | | | |
| Study  (Author/Year of publication) | | | Study design  (P/R) ^a^ | | | Sample size | | IMRT  (Y/N) | | MRI staging  (Y/N) | | TNM/UICC staging | | | Prognostic factor | | Survival outcomes **^b^** | | | | | | | | | | | | Quality score ^c^ | | | | | Proposed changes | |
|  | | |  | | |  | |  | |  | |  | | |  | | OS | | PFS | | | DMFS | | | LRFS | | | |  | | | | |  | |
| **(A) Retropharyngeal LN (RPLN)** | | | | | | | | | | | | | | | | | | | | | | | | | | | | | | | | | | | |
| Shi (2014)^31^ | | | R | | | 142 | | Y | | Y | | 7^th^ Edition  N1 disease | | | RLN vs. CLN | | Y | | Y | | | Y | | | Y | | | | 5 | | | | | Better prognosis: CLN or RLN-only > CLN + RLN | |
| Tang (2014)^32^ | | | R | | | 749 | | Y | | Y | | 7^th^ Edition  Stage I–IVB | | | RLN | | Y | | Y | | | Y | | | Y | | | | 5 | | | | | Better prognosis: RLN-only > Other N1 disease; No difference in unilateral vs. bilateral RLN | |
| Huang L, Zhang Y, Liu Y (2019)^33^ | | | R | | | 1225 | | Y | | Y | | 8^th^ Edition  Stage I–IVB | | | RLN | | Y | | Y | | | Y | | | N | | | | 6 | | | | | Upgrading bilateral RLN metastasis from N1 to N2 | |
| **(B) Parotid LN (PLN)** | | | | | | | | | | | | | | | | | | | | | | | | | | | | | | | | | | | |
| Xu (2017)^34^ | | | R | | | 1616 | | Y | | Y | | 7^th^ Edition  Stage I–IVB | | | PLN | | Y | | Y | | | Y | | | Y | | | | 6 | | | | | PLN similar prognosis as that of N3 | |
| Zhang (2019)^35^ | | | R | | | 10126 | | Y | | Y | | 8^th^ Edition  Stage I–IVB | | | PLN | | N | | Y | | | Y | | | Y | | | | 5 | | | | | PLN similar prognosis as that of N3 | |
| **(C) Extranodal extension (ENE)** | | | | | | | | | | | | | | | | | | | | | | | | | | | | | | | | | |  | |
| Guo (2015)^38^ | | | R | | | 1197 | | Y | | Y | | 7^th^ Edition  Stage I–IVB | | | ENE and LNN | | Y | | N | | | Y | | | Y | | | | 6 | | | | | ENE and LNN are not prognostic | |
| Ai (2019)^36^ | | | R | | | 546 | | Y | | Y | | 8^th^ Edition  Stage I–IVB | | | ENE | | Y | | N | | | Y | | | Y | | | | 5 | | | | | Grade 2 ENE (muscle/skin/salivary gland) classified as N3 | |
| Lu (2019)^37^ | | | R | | | 1616 | | Y | | Y | | 8^th^ Edition  Stage II | | | ENE | | Y | | N | | | Y | | | Y | | | | 5 | | | | | ENE is associated with poorer prognosis | |
| **(D) Lymph node necrosis (LNN)** | | | | | | | | | | | | | | | | | | | | | | | | | | | | | | | | | | | |
| Guo (2015)^38^ | | | R | | | 1197 | | Y | | Y | | 7^th^ Edition  Stage I–IVB | | | ENE and LNN | | Y | | N | | | Y | | | Y | | | | 6 | | | | | ENE and LNN are not prognostic | |
| Lan (2015)^39^ | | | R | | | 1800 | | N | | Y | | 7^th^ Edition  Stage I–IVB | | | LNN | | Y | | Y | | | Y | | | Y | | | | 4 | | | | | N1 with LNN = N2 no LNN  N2 with LNN = N3 | |
| Luo Y, Ren J, Zhou P (2016)^40^ | | | R | | | 189 | | Y | | Y | | 7^th^ Edition  Stage III–IVB | | | LNN | | Y | | Y | | | Y | | | Y | | | | 5 | | | | | N1 with LNN = N2 no LNN  N2 with LNN = N3 | |
| Lu L, Wei X, Li YH (2017)^41^ | | | R | | | 252 | | Y | | Y | | 7^th^ Edition  Stage II–IVB | | | LNN | | Y | | Y | | | Y | | | Y | | | | 4 | | | | | No suggestion | |
| Feng (2019)^42^ | | | R | | | 616 | | Y | | Y | | 8^th^ Edition  Stage I–IVB | | | LNN | | Y | | N | | | Y | | | Y | | | | 6 | | | | | N1 with LNN = N2 no LNN  N2 with LNN = N3 | |
| Ting (2017)^44^ | | | R | | | 257 | | NA | | Y | | 7^th^ Edition  Stage I–IVB | | | Cystic lymph node metastasis (CLNM) | | Y | | Y | | | N | | | N | | | | 5 | | | | | CLNM can categorize N2 patients into two prognostic groups | |
| **(E) Simplification of N-classification** | | | | | | | | | | | | | | | | | | | | | | | | | | | | | | | | | | | |
| Yue (2014)^47^ | | | R | | | 749 | | Y | | Y | | 7^th^ Edition  Stage I–IVB | | | SCF LN, LN >6 cm | | N | | Y | | | Y | | | N | | | | 6 | | | | | Merge N3a and N3b into N3 | |
| Kang (2016)^22^ | | | R | | | 492 | | Y | | Y | | 7^th^ Edition Stage I–IVB | | | N1 vs. N2 vs. N3 | | Y | | N | | | Y | | | N | | | | 6 | | | | | Merge N3a and N3b into N3 | |
| Pan (2016)^14^ | | | R | | | 1609 | | Y | | Y | | 7^th^ Edition  Stage I–IVB | | | SCF LN, LN >6 cm | | Y | | N | | | Y | | | Y | | | | 5 | | | | | Merge N3a and N3b into N3 | |
| Liang (2016)^23^ | | | R | | | 752 | | Y | | Y | | 7^th^ Edition  Stage I–IVB | | | N1 vs. N2 vs. N3 | | Y | | N | | | Y | | | N | | | | 4 | | | | | Merge N1 and N2 | |
| **(F) Others** | | | | | | | | | | | | | | | | | | | | | | | | | | | | | | | | | | | |
| Jiang (2017)^43^ | | | R | | | 406 | | Y | | Y | | 7^th^ Edition  N1–3M0 | | | Posterior to Level V (PLV) LN metastasis | | Y | | Y | | | Y | | | Y | | | | 3 | | | | | PLV LN should be defined as a new lymph node segment | |
| Kang (2018)^45^ | | | P | | | 492 | | Y | | Y | | 7^th^ Edition  Stage I–IVB | | | Cervical LN level | | Y | | Y | | | Y | | | Y | | | | 6 | | | | | N1 [RLN or/and unilateral upper cervical (I, II, III, Va, VIIb, VIII, IX, and X regions) LNs, N2  (bilateral upper cervical LN) and N3 (LN in IVa and Vb regions and their lower regions) | |
| Zhou (2018)^46^ | | | R | | | 354 | | Y | | Y | | 8^th^ Edition  Stage I–IVB | | | Number of LN regions (LNR) | | Y | | Y | | | Y | | | N | | | | 6 | | | | | New N classification based on LNR: 0–1, 2–6, ≥7 | |
| **(III) M-classification (n=5)** | | | | | | | | | | | | | | | | | | | | | | | | | | | | | | | | | | | |
|  | |  | | |  | | | |  | |  | | |  | |  | | | | | **Survival outcome** ^b^ | | | | | | | | | |  |  | | | |
| Study  (Author/Year of publication) | | Study design  (P/R) ^a^ | | | Sample size | | | | IMRT  (Y/N) | | MRI staging  (Y/N) | | | TNM/UICC staging | | Prognostic factor | | | | | OS | | | PFS | | | | DMFS | | LRFS | Quality score ^c^ | Changes proposed by authors | | | |
| Shen LJ, Wang SY, Xie GF (2015)^49^ | | R | | | 505  (synchronous) | | | | N | | Y | | | 7^th^ Edition  Stage IVC | | Recategorization of M stage, as proposed by authors | | | | | Y | | | N | | | | N | | N | 4 | Recategorization of M stage:  M1a: single lesion to isolated organ (except for the liver)  M1b: single lesion to the liver, or multiple lesions in other organs  M1c: multiple lesions in the liver | | | |
| Shen (2016)^48^ | | R | | | 1172  (metachronous) | | | | N | | Y | | | 7^th^ Edition  Stage IVC | | Subdivision of M1 stage, as proposed by authors | | | | | Y | | | N | | | | N | | N | 5 | Subdivision of M1 stage:  M1a, a single lesion in a single organ or location  M1b, multiple lesions in a single organ or location; and  M1c, metastases in multiple locations | | | |
| Jiang (2016)^50^ | | R | | | 347 (synchronous) | | | | Y | | Y | | | 7^th^ Edition  Stage IVC | | M1b vs. M1a of their newly proposed ten-signature classifier | | | | | Y | | | N | | | | N | | N | 5 | To use ten-signature classifier * as a classifier for M1a and M1b. | | | |
| Zou (2017)^52^ | | R | | | 462 | | | | N | | Y | | | 7^th^ Edition  Stage IVC | | Number of metastatic lesions and position of lesions, namely, liver | | | | | Y | | | N | | | | N | | N | 4 | M1a: oligometastasis without liver involvement  M1b, multiple metastases without liver involvement  M1c, liver involvement irrespective of metastatic lesions. | | | |
| Tian (2016)^51^ | | R | | | 263 | | | | N | | Y | | | 7^th^ Edition  Stage IVC | | Number of metastatic lesions (M1b vs. M1a) | | | | | Y | | | N | | | | N | | N | 5 | M1a: 5 single-organ metastases or 1–5 lesions  M1b: 5 multiorgan metastases or >6 lesions | | | |

***** Ten-signature classifier: oligometastasis, extraregional LN metastases, N-stage, EB-VCA IgA, neutrophil count, platelet count, hemoglobin, glutamic-pyruvic transaminase, glutamyl transpeptidase, and monocyte count

^a^ Study design: P, prospective; R, retrospective. ^b^ survival outcomes: OS, overall survival; PFS, progression-free survival; DMFS, distant-metastasis free survival; LRFS, local-relapse free survival; LN, lymph node; IMRT-intensity-modulated radiation therapy; UICC, Union for International Cancer Control. ^c^ quality score: 1=poor, 6=excellent.

# Supplementary Table 3. Characteristics of studies on the correlation of the EBV DNA level and survival outcomes (n=22)

| Study |  |  |  |  |  | Cutoff value (copies/mL) ^b^ | | | Survival outcome ^c^ | | | |  |  |
| --- | --- | --- | --- | --- | --- | --- | --- | --- | --- | --- | --- | --- | --- | --- |
| Author/Year of publication | Study design  (P/R) ^a^ | Sample size | TNM staging  UICC staging edition | EBV DNA extraction tool | EBV DNA genome | Pre | Mid | Post | OS | PFS | DMFS | LRFS | Treatment plan | Quality score  1 = poor; 6 = excellent |
| Chen M, Yin L, Wu J (2015)^53^ | P | 165 | M0  III–IVb (7^th^) | QIAamp DNA Blood Mini Kit | BamH I-W region | NR | - | - | Y | Y | - | - | IMRT + chemo | 5 |
| Tang (2015)^54^ | P/R | 6337 | M0  I–IV (7^th^) | QIAamp Blood Kit | BamH I-W region  EBNA-1 region | 4000 | - | - | Y | Y | Y | - | RT ± chemo | 4 |
| Yang (2015)^55^ | R | 1168 | T1–3N0–3M0 | - | - | 3760 | - | - | Y | - | Y | - | RT ± chemo | 4 |
| Zhao (2015)^56^ | R | 637 | I–IV (7^th^) | QIAamp Blood Kit | BamH I-W region  GAPDH (house  keeping gene) | 1500 | - | NR | Y | - | Y | - | RT ± induction chemo | 4 |
| Chen (2016)^57^ | R | 404 | M0  III–IVb (7^th^) | TaqMan PCR Core Reagent Kit | BamH I-W region  EBNA-1 region | 4000 | - | - | Y | Y | Y | - | IMRT + cisplatin-based chemo | 4 |
| Du (2016)^58^ | R | 296 | T1–2N0–1 | - | BamH I-W region | 4000 | - | - | - | - | Y | - | IMRT ± (NACT or CCRT) | 6 |
| Lv (2016)^59^ | R | 1501 | M0  I–III (7^th^) | - | - | 4000 | - | - | Y | Y | Y | Y | IMRT ± chemo | 3 |
| Peng H, Chen L, Zhang Y (2016)^117^ | R | 1106 | T1–4N0–3M0  III–IVb (7^th^) | QIAamp Blood Kit | BamH I-W region | 0 | - | - | Y | Y | Y | - | IMRT ± NACT/ACT or CCRT ± NACT/ACT | 3 |
| Peng H, Guo R, Chen L (2016)^60^ | R | 584 | I–IVb (7^th^) | QIAamp Blood Kit | BamH I-W region | 2010 | - | 20 | Y | Y | - | Y | IMRT ± induction or CCRT | 4 |
| Zhang (2016)^57^ | R | 1467 | M0  I–IVb (7^th^) | - | - | 4000 | - | - | Y | Y | Y | Y | IMRT | 6 |
| Jin YN, Yao JJ, Zhang F (2017)^61^ | R | 1036 | T1–4N0–3  III–IVb (7^th^) | QIAamp DNA Blood Minikit | BamH I-W region | 1500 | - | - | Y | Y | Y | Y | IMRT ± chemo | 4 |
| Yao (2017)^62^ | R | 787 | N1M0 | QIAamp Blood Kit | BamHI‐W region | 4000 | - | - | - | - | Y | - | IMRT ± NA/CCRT | 4 |
| Chen (2018)^63^ | R | 385 | T1–2N0–1M0  II (7^th^) | - | - | 0 | - | - | - | Y | Y | Y | IMRT or CCRT | 5 |
| He 2018^64^ | R | 949 | T1–4N0–3  I–IVb (8^th^) | - | BamHI-W fragment region | 2500 | 871 | 721 | Y | Y | Y | - | IMRT ± chemo | 5 |
| Peng (2018)^65^ | R | 3794 | I–IVb (8^th^) | QIAamp Blood Kit | BamH I-W region | 0 | - | - | Y | Y | Y | - | IMRT ± chemo | 5 |
| Du (2019)^66^ | R | 607 | N1–3M0  II–IV (8^th^) | - | BamH I-W region | 4000 | - | - | Y | Y | Y | Y | IMRT ± chemo | 4 |
| Guo (2019)^67^ | R | 979 | M0  I–IV (8^th^) | QIAamp Blood Kit | BamH I-W region | n/a | - | - | Y | Y | Y | - | IMRT ± chemo | 4 |
| Huang CL, Sun ZQ, Guo R (2019)^68^ | R | 278 | III–IV (8^th^) | QIAamp Blood Kit | BamH I-W region | 7000 | - | 0 | Y | Y | Y | Y | ICT ± CCRT | 5 |
| Lee (2019)^71^ | R | 518 | I–IV (8^th^) | QIAamp Blood Kit | BamH I-W region | 500 | - | - | Y | Y | Y | Y |  | 3 |
| Sun XS, Chen WH, Liu SL (2019)^70^ | R | 2742 | T1–3N0–2  II–III (8^th^) | - | BamH I-W region | 1460 | - | - | Y | Y | Y | Y | IMRT ±- CCRT | 4 |
| Sun XS, Liang YJ, Liu SL (2019)^69^ | R | 226 | M1 | - | - | - | - | 25000 | Y | - | - | - | PCT ± IMRT/LRRT | 3 |
| Sun XS, Liu LT, Liu SL (2019)^69^ | R | 502 | M1 | - | - | 0 | - | - | Y | - | - | - | PCT ± LRRT | 4 |

^a^ Study design: prospective (P)/retrospective (R). ^b^ Cutoff values: pretreatment (Pre), midtreatment (Mid), or posttreatment (Post). Survival outcomes: OS, overall survival; PFS, progression-free survival; DMFS, distant-metastasis free survival; LRFS, local-relapse free survival; IMRT, intensity-modulated radiation therapy; CCRT, concurrent chemoradiotherapy; UICC, Union for International Cancer Control, 7^th^ Edition;

# Supplementary Table 4. Characteristics of studies on the correlation of tumor volume and survival outcomes (n=12)

| Study |  |  |  | Cutoff value (cm^3^) | | Survival outcome ^b^ | | | |  |  |  |
| --- | --- | --- | --- | --- | --- | --- | --- | --- | --- | --- | --- | --- |
| Author/Year of publication | Study design  (P/R) ^a^ | Sample size | TNM staging | Primary tumor volume | Nodal volume | OS | PFS | DMFS | LRFS | Treatment plan | Survival probabilities | Quality score  1 = poor; 6 = excellent |
| Tian (2015)^72^ | R | 229 | Stage I–Iva  (2009AJCC) | 38 | NA | Y | - | - | - | Concurrent, induction and concurrent + induction (For recurrent T3–T4) | 5-year:  OS: 48.7 vs 15.2% | 4 |
| He 2016^73^ | R | 358 | T3–4N0–3M0  (AJCC7) | 46.4 | NA | Y | Y | Y | Y | RT-only, concurrent, adjuvant and neoadjuvant | 3-year  OS: 90.5% vs. 75.5%  DFS: 85.3% vs. 67.5%  DMFS: 90.2% vs. 74.5%  LRFS: 96.6% vs. 90.9% | 5 |
| Lu (2016)^74^ | P | 180 | Stage I–IVb  (2002UICC) | 20 | 10 | Y | Y | - | - | Stage I–II: RT-only  Stage III–IVb: Concurrent w/wo Induction | 5-year (GTVnx)  DFS: 81.8% vs. 62.3%  OS: 95.1% vs. 70.6%  5-year (GTVnd)  DFS: 76.0% vs. 54.5%  OS: 86.0% vs. 75.8% | 4 |
| Qin (2016)^76^ | P | 249 | Stage III–IVb (2002AJCC) | 33 | NA | Y | - | Y | Y | Concurrent | 5-year  OS: 86.1% vs. 70.5%  DMFS: 85.1% vs 70.5% | 3 |
| Luo Y, Gao Y, Yang G (2016)^75^ | R | 110 | 7^th^ Edition  T4-only | NA | 14.1 | Y | Y | Y | - | CCRT ± ICT | 5-year: DMFS 79.0% vs. 56.1% (𝑃 = 0.007)  PFS: 73.1% vs, 55.0% (𝑃 = 0.021)  OS: 75.6% vs. 53.0% (𝑃 = 0.028) | 4 |
| Chen (2017)^77^ | R | 1230 | Stage II–IVb  (AJCC7) | NA | 7.2/35.7 | Y | - | Y | - | RT-only → Stage I  Concurrent → Stage II  Concurrent w/wo neo/neoadjuvant/both → Stage III–IVb | 5-year  OS: 90.2% vs. 88.2% vs. 62.3%  DFS:82.5% vs. 78.2% vs. 56.5%  RRFS: 94.4% vs. 94.4% vs. 87.3%  DMFS: 92.3% vs. 87.1% vs. 66.8% | 6 |
| Liu T, Lv J, Qin Y (2017)^81^ | P | 143 | Stage III–IVb (UICC 7) | 43.5 | 15.0 | Y | Y | Y | Y | Concurrent | No | 5 |
| Zhang (2017)^78^ | R | 393 | All (AJCC7) | 23 | NA | Y | Y | Y | Y | RT-only → Stage I, Concurrent w/wo neoadjuvant/adjuvant | 5-year | 3 |
| Liang (2017)^79^ | R | 455 | M0  AJCC7 | 28 | - | Y | - | Y | Y | IMRT ± cisplatin-based chemotherapy | 4-year OS: 95.1% vs. 75.4% (*P* < 0.001)  4-year FFS: 93% vs. 71.4% (*P* < 0.001)  4-year DMFS: 94.5% vs. 79.4% (*P* < 0.001)  4-year LRFS: 96.2% vs. 88% (*P* = 0.001) | 4 |
| Chen (2018)^63^ | P | 385 | Stage II (AJCC7) | 30 | - | Y | Y | Y | Y | RT-only, Concurrent |  | 4 |
| Jeong (2018)^80^ | R | 133 | All (AJCC7 & UICC7 & 8^th^ Edition) | 33 | - | Y | - | Y | Y | Concurrent, Induction, Adjuvant | 5-year OS | 5 |
| Peng (2018)^65^ | R | 3794 | All (AJCC8) | NA | NA | Y | Y | Y | Y | RT w/wo Concurrent | 3-year | 5 |

^a^ Study design: prospective (P)/retrospective (R). ^b^ Survival outcomes: OS, overall survival; PFS, progression-free survival; DMFS, distant-metastasis-free survival; LRFS, local-relapse-free survival; RT, radiation therapy; IMRT, intensity-modulated radiation therapy; CCRT, concurrent chemoradiotherapy AJCC7, American Joint Committee on Cancer, 7th Edition; UICC7, Union for International Cancer Control, 7th Edition; ICT, induction chemotherapy.

# Supplementary Table 5. Characteristics of studies on the correlation of plasma biomarkers and survival outcomes (n=18)

| Study |  |  |  |  |  | Survival outcome ^c^ | | | |  |  |
| --- | --- | --- | --- | --- | --- | --- | --- | --- | --- | --- | --- |
| Author/Year of publication | Study design  (P/R) ^a^ | Sample size | TNM staging | Type of biomarkers^b^ | Cutoff value | OS | PFS | DMFS | LRFS | Treatment plan | Quality score  1 = poor; 6 = excellent |
| Tang (2015)^54^ | P+R | 6337 | T1–4N0–3 | hs-CRP | 3 mg/L | Y | Y | Y | - | RT-only, Concurrent | 6 |
| Chen YP, Zhao BC, Chen C (2015)^82^ | R | 2626 | M0  (AJCC7) | PLT | 300 × 10^9^/L | Y | Y | Y | Y | RT ± Chemotherapy (radical) | 5 |
| Tao (2016)^98^ | R | 719 | T1–4N0–3M0 (AJCC7) | CRP/Alb ratio | 0.141 | Y | - | - | - | RT w/wo Concurrent/Induction/Adjuvant (maybe multiple) | 4 |
| Li JP, Chen SL, Liu XM (2016)^83^ | R | 409 | AJCC6 | CRP/Alb ratio  NLR  PLR | 0.03  2.48  146.2 | Y | Y | Y | Y | CCRT/RT | 6 |
| Li X, Chang H, Tao Y (2016)^84^ | R | 249 | AJCC7 | Hb | 13.0 g/dL (male)  12.0 g/dL (female) | - | - | - | - | CCRT/RT | 6 |
| Lu AY, Li HF, Zheng YM (2019)^85^ | R | 140 | Stage I–IVa (Chinese 2008 staging system) | NLR, LMR, and PLR | NLR: 2.28  LMR: 2.26  PLR: 174 | Y | - | - | - | RT w/wo Chemotherapy | 5 |
| Xie (2017)^86^ | R | 168 | Stage I–IVB (AJCC7) | PDW and PLT | PDW: 16.3 fL  PLT: 266×10^9^/L | Y | - | - | - | Stage I–II: RT w/wo Concurrent  Stage III–IVb: Concurrent w/wo Induction/Adjuvant | 6 |
| Zhang (2017)^87^ | R | 1302 | Stage I–IV (AJCC7) | Hb | 12.0 g/dL (male)  11.0 g/dL (female) | Y | Y | Y | Y | Stage I: Concurrent  Stage II–IV: Concurrent w/wo Adjuvant/Neoadjuvant | 4 |
| Zhou (2017)^88^ | R | 339 | Stage I–IV  (AJCC6) | LDH  ALP | 220 U/L | Y | - | Y | - | RT ± chemo (CCRT/ICT/ACT) | 3 |
| Wang Y, Yang L, Xia L (2018)^89^ | R | 1168 | Stage I–IV (AJCC) | CRP/Alb ratio | 0.081 | - | - | Y | - | Stage I–II: RT-only  Stage III–IV: Induction/Adjuvant | 5 |
| Wang YQ, Chen YP, Zhang Y (2018)^90^ | R | 1490 | Stage I–IV (AJCC8) | TIL | 245 | Y | Y | Y | Y | RT w/wo chemo | 5 |
| Ye (2018)^91^ | R | 427 | Stage I–IV (AJCC7) | NLR and PLR | NLR: 2.32  PLR: 123 | Y | Y | - | - | RT-only, Concurrent w/wo Neoadjuvant/Adjuvant | 4 |
| Akcay (2019)^92^ | R | 62 | Stage I–IVb (AJCC/UICC7) | NLR | 3 | Y | - | - | - | T1–T2N0: RT-only  Other: Concurrent w/wo Adjuvant | 5 |
| Gundog (2019)^93^ | R | 95 | Stage II–IVA (AJCC8) | AGR, prognostic nutrition index | AGR: 1.19  PNI: 45.45 | Y | - | Y | Y | Chemoradiotherapy (No type mentioned) | 6 |
| He (2019)^94^ | R | 511 | Stage I–IV (AJCC8) | Plasma D-dimer, Alb | Plasma D-dimer: 0.675  Alb: 45 | Y | - | Y | - | Stage I: RT-only  Stage II: Concurrent  Stage III–IVab: Concurrent w/wo Neoadjuvant/Adjuvant | 6 |
| Long (2019)^95^ | R | 172 | Stage I–IV (AJCC7) | LDH | 229U/L | Y | - | Y | - | Stage I: Mostly RT-only  Stage II–IV: Concurrent w/wo Neoadjuvant/Adjuvant, Neoadjuvant/Adjuvant | 5 |
| Topkan (2019)^96^ | R | 149 | Stage II–IVb (AJCC7) | Hb | 11.0 g/dL | Y | Y | - | Y | Concurrent | 5 |
| Yao (2019)^97^ | R | 1550 | Stage II–IVa (AJCC8) | NLR | 2.50 | Y | Y | Y | Y | Stage II: Concurrent  Stage III–IVa: Concurrent w/wo Neoadjuvant/Adjuvant | 6 |

^a^ Study design: prospective (P)/retrospective (R). ^b^ Types of markers: AGR, albumin–globulin ratio; Alb, albumin; ALP, alkaline phosphatase; CRP, C-reactive protein; hs-CRP, high-sensitivity C-reactive protein; Hb, hemoglobin; LDH, lactate dehydrogenase; LMR, lymphocyte-to-monocyte ratio; NLR, neutrophil-to-lymphocyte ratio; PDW, platelet distribution width; PLR, platelet-to-lymphocyte ratio; PLT, platelet; TIL, tumor-infiltrating lymphocytes. ^c^ Survival outcomes: DMFS, distant-metastasis-free survival; LRFS, local-relapse-free survival; OS, overall survival; PFS, progression-free survival; RT, radiation therapy; IMRT, intensity-modulated radiation therapy; CCRT, concurrent chemoradiotherapy, AJCC7, American Joint Committee on Cancer, 7th Edition; UICC7, Union for International Cancer Control, 7th Edition; ICT, induction chemotherapy; ACT, adjuvant chemotherapy. HR, hazard ratio; CI, confidence interval

# Supplementary Table 6. Characteristics of studies on the correlation of PET parameters and survival outcomes (n=15)

| Study |  |  |  |  |  | Survival outcome ^c^ | | | |  |  |
| --- | --- | --- | --- | --- | --- | --- | --- | --- | --- | --- | --- |
| Author/Year of publication | Study design  (P/R) ^a^ | Sample size | TNM staging  UICC staging | Nature of PET parameters | Cutoff value of PET parameters | OS | PFS | DMFS | LRFS | Treatment plan | Quality score  1 = poor; 6 = excellent |
| Yoon (2014)^99^ | R | 53 | AJCC (version not specified)  M0 | SUV_max_  MTV_2.5_  MTV_3.0_ | ≥8.9  ≥31.45 cm^3^  ≥23.01 cm^3^ | Y | - | - | - | NACT (docetaxel/5-FU) + RT  CCRT for advanced | 3 |
| Zaghloul (2014)^100^ | R | 70 | AJCC6  T1N1–3  T2–4N0–3 (LANPC) | SUV_max_ | ≥8.0 | Y | Y (DFS) | - | - | ICT (TPF) ± CCRT | 3 |
| Hsieh (2015)^101^ | R | 174 | AJCC7 | SUV_max_ | ≥8.35 | Y | - | Y | Y | CCRT ± ACT | 4 |
| Shen T, Tang LQ, Luo DH (2015)^102^ | R | 194 (107: LR; 87: DM) | AJCC7  I–IV | SUV_max_ | ≥8.65 (LR)  ≥13.55 (DM) | Y | - | - | - | ACT (platinum-based) | 4 |
| Xiao W, Xu A, Han F (2015)^103^ | R | 179 | AJCC6 | SUV_max_ | ≥10.22 | Y | - | Y | - | IMRT ± CCRT (cisplatin, advanced-only) | 5 |
| Yoon (2016)^104^ | R | 97 | AJCC7  III–IVa/b | TLG | 322.7 | Y | Y | Y | Y | 3D-CRT/IMRT + CCRT ± ICT | 6 |
| Zhang (2016)^105^ | R | 449 | AJCC7 | SUV_max_-T (SUV_max_, primary tumour)  SUV_max_-N (SUV_max_, cervical lymph nodes) | 10.45  6.65 | - | - | Y | - | IMRT (I) ± CCRT ± ACT/NCT (II–IVb) | 4 |
| Jeong (2017)^106^ | R | 145 | AJCC7  II–IVb | SUV_max_-T  SUV_mean_-T  SUV_peak_-T  SUV_max_-N  SUV_peak_-N | 8.0  3.2  10.2  10.6  7.7 | - | - | Y | - | IMRT + (CCRT ± ICT) | 6 |
| Jin YN, Yao JJ, Wang SY (2017)^107^ | R | 1811 | AJCC7  II–IVb | SUV_max_-T  SUV_max_-N | 8.95  5.75 | - | - | Y | - | IMRT + CCRT ± ACT (III–IVb) | 6 |
| Lee (2017)^108^ | R | 53 | AJCC7  M0 | SUV_max_-T  SUV_max_-N (met LN) | 13.4 | Y | Y | Y | Y | IMRT + CCRT | 5 |
| Liu F, Xi XP, Wang H (2017)^109^ | R | 213 | AJCC7  III–IVb | PET-CT-guided dose-painting IMRT vs CT-based IMRT | - | Y | Y | Y | Y | PET-CT-guided DP-IMRT (n=101)  CT-based IMRT (n=112) | 5 |
| Zhong (2017)^110^ | R | 121 | AJCC7 | SUV_max_-T  SUV_max_-N | 12.35  10.15 | - | Y | Y | Y |  | 5 |
| Alessi (2019)^111^ | R | 160 | AJCC7  II–IVb | SUV_max_-T  SUV_mean_-T  TLG | 18.8  9.5  382.2 | Y | Y | - | - | IMRT (IIa) ± chemo (IIb–IV) | 3 |
| Fei (2019)^112^ | R | 82 | AJCC8 | PTV  SUV_max_  SUV2.5  SUV3.0  SUV3.5  SUV4.0  SUV4.5  SUV5.0  SUV5.5  SUV6.0 | 48.70  8.20  14.62  8.77  7.26  5.73  4.42  3.30  2.56  6.02 | - | Y | - | - | IMRT (I) ± CCRT (II–IVb) ± NACT (III–IV) | 3 |
| Sun XS, Liang YJ, Liu SL (2019; BMC Cancer)^113^ | R | 253 | AJCC8  M1 | SUV_max_-T, SUV_max_-N  SUV_max_-M | 17.0  12.7  6.9 | Y | - | - | - | Cisplatin-based palliative chemo | 5 |

^a^ Study design: prospective (P)/retrospective (R). ^b^ Survival outcomes: OS, overall survival; PFS, progression-free survival; DMFS, distant-metastasis-free survival; LRFS, local-relapse-free survival.

Abbreviations: HR, hazard ratio; CI, confidence interval; SUV_max_, maximum standardized uptake value; SUV_mean_, mean standardized uptake value; MTV, metabolic tumor volume; TLG, total lesion glycolysis; T, primary tumor; N, lymph node; M, metastasis; LR, local recurrence; RT, radiation therapy; IMRT, intensity-modulated radiation therapy; CCRT, concurrent chemoradiotherapy, AJCC7, American Joint Committee on Cancer, 7^th^ Edition; UICC7, Union for International Cancer Control, 7^th^ Edition; ICT, induction chemotherapy; ACT, adjuvant chemotherapy; DP-IMRT, dose-painted intensity-modulated radiation therapy; 3D-CRT, 3D conformal radiation therapy; NACT, neo-adjuvant chemotherapy.
